# Effect of a Brown Rice Based Vegan Diet and Conventional Diabetic Diet on Glycemic Control of Patients with Type 2 Diabetes: A 12-Week Randomized Clinical Trial

**DOI:** 10.1371/journal.pone.0155918

**Published:** 2016-06-02

**Authors:** Yu-Mi Lee, Se-A Kim, In-Kyu Lee, Jung-Guk Kim, Keun-Gyu Park, Ji-Yun Jeong, Jae-Han Jeon, Ji-Yeon Shin, Duk-Hee Lee

**Affiliations:** 1 Department of Preventative Medicine, School of Medicine, Kyungpook National University, Daegu, Korea; 2 Department of Biomedical Science, Kyungpook National University, Daegu, Korea; 3 BK21 Plus KNU Biomedical Convergence Program, Department of Biomedical Science, Kyungpook National University, Daegu, Korea; 4 Department of Endocrinology, School of Medicine, Kyungpook National University, Daegu, Korea; 5 Department of Preventive Medicine, School of Medicine, Eulji University, Daejeon, Republic of Korea; McMaster University, CANADA

## Abstract

**Objective:**

Several intervention studies have suggested that vegetarian or vegan diets have clinical benefits, particularly in terms of glycemic control, in patients with type 2 diabetes (T2D); however, no randomized controlled trial has been conducted in Asians who more commonly depend on plant-based foods, as compared to Western populations. Here, we aimed to compare the effect of a vegan diet and conventional diabetic diet on glycemic control among Korean individuals.

**Materials and Methods:**

Participants diagnosed with T2D were randomly assigned to follow either a vegan diet (excluding animal-based food including fish; n = 46) or a conventional diet recommended by the Korean Diabetes Association 2011 (n = 47) for 12 weeks. HbA1c levels were measured at weeks 0, 4, and 12, and the primary study endpoint was the change in HbA1c levels over 12 weeks.

**Results:**

The mean HbA1c levels at weeks 0, 4, and 12 were 7.7%, 7.2%, and 7.1% in the vegan group, and 7.4%, 7.2%, and 7.2% in the conventional group, respectively. Although both groups showed significant reductions in HbA1C levels, the reductions were larger in the vegan group than in the conventional group (-0.5% vs. -0.2%; p-for-interaction = 0.017). When only considering participants with high compliance, the difference in HbA1c level reduction between the groups was found to be larger (-0.9% vs. -0.3%). The beneficial effect of vegan diets was noted even after adjusting for changes in total energy intake or waist circumference over the 12 weeks.

**Conclusion:**

Both diets led to reductions in HbA1c levels; however, glycemic control was better with the vegan diet than with the conventional diet. Thus, the dietary guidelines for patients with T2D should include a vegan diet for the better management and treatment. However, further studies are needed to evaluate the long-term effects of a vegan diet, and to identify potential explanations of the underlying mechanisms.

**Trial Registration:**

CRiS KCT0001771

## Introduction

A healthy diet is one of the core elements in the management of type 2 diabetes (T2D), along with regular exercise and pharmacotherapy [1]. In fact, diet plays important roles in T2D prevention and management, such as decreasing the risk of diabetes in individuals with obesity and pre-diabetes and avoiding the associated complications [2]; however, there is currently no general optimal meal plan or dietary pattern for T2D patients [3].

A vegetarian or vegan diet has been suggested to be clinically beneficial in the management of diabetes [4, 5]. Interestingly, a randomized clinical trial (RCT) has suggested that a low-fat vegan diet can be more effective in glycemic and lipid control than a conventional diet recommended by the American Diabetes Association (ADA) [6].

Thus far, most of the RCTs on vegetarian or vegan diets in T2D patients have included populations from Western countries [5]. Compared to the populations from Western countries, Asians mainly consume a diet based on plant foods such as rice, vegetables, and fresh fruits [7]. Hence, the use of a vegan diet in Asians with T2D may be more effective than that in Western populations.

In addition, both epidemiological and experimental evidence has suggested that the chronic exposure to chemicals such as persistent organic pollutants (POPs) may disturb glucose and lipid metabolism [8]. As fatty animal foods, including fish, are the main sources of exposure to POPs [9], the use of a vegan diet that excludes any animal foods could also be beneficial in terms of avoiding POP exposure.

In the present study, we conducted an RCT to determine the effect of a vegan diet on glycemic control and other cardiovascular risk factors in Korean patients with T2D.

## Materials and Methods

### Participants

Participants were recruited through advertisements in the endocrinology outpatient clinic of Kyungpook National University Hospital, Hypertension-Diabetes Education Center, and 4 public health centers in Daegu city from March 2012 through August 2012. The inclusion criteria were as follows: age of 30–70 years; use of hypoglycemic medications for ≥6 months; and HbA1c level of 6.0–11.0%. The exclusion criteria were as follows: increased dose of hypoglycemic medication or the addition of a new drug in the regimen during the last 2 months; current vegetarian status; pregnancy; or severe complications such as chronic renal failure.

### Sample size estimation

Considering the two-sample t-test for a between-group HbA1c difference (effect size) of 0.65%, standard deviation of 1.0, α level of 0.05 for a two-tailed test, power of 80%, and a loss of follow-up rate of 30%, we found that 53 participants would be required for each group [10]. Among the 106 participants enrolled from April 2012 through August 2012, 53 subjects and 53 subjects were stratified according to a cutoff HbA1c value of 8.0% and randomly allocated to the conventional Korean Diabetic Association (KDA) diet group and vegan diet group, respectively, using stratified block randomization with a block size of 4.

### Intervention

#### 1) Experimental group: vegan diet

Participants were asked to follow a vegan diet consisting of whole grains, vegetables, fruit, and legumes. The following instructions were provided to these patients: 1) ingest unpolished rice (brown rice); 2) avoid polished rice (white rice); 3) avoid processed food made of rice flour or wheat flour; 4) avoid all animal food products (i.e., meat, poultry, fish, daily goods, and eggs); and 5) favor low-glycemic index foods (e.g., legumes, legumes-based foods, green vegetables, and seaweed). Participants were carefully educated on the foods they should consume and should avoid. The amount and frequency of food consumption, energy intake, and portion sizes were not restricted over the 12-week period.

#### 2) Control group: conventional diet

The conventional diet followed the treatment guidelines for diabetes recommended by the KDA 2011 [11]. Participants were asked to 1) restrict their individualized daily energy intake based on body weight, physical activity, need for weight control, and compliance; 2) total calorie intake comprised 50–60% carbohydrate, 15–20% protein (if renal function is normal), <25% fat, <7% saturated fat, minimal trans-fat intake, and ≤200 mg/day cholesterol. A dietitian estimated the individual daily energy requirement (standard body weight [kg] × 30–35 [kcal/kg]) while considering moderate physical activity in the participants, and established the food exchange lists based on the individual daily energy requirements as per the KDA 2011 guidelines. The type and amount of food were classified into 6 food categories (grains, meat, vegetables, fats and oils, milk, and fruits) based on the food exchange lists, and were prescribed to all participants in the KDA group. The daily energy requirement and daily proportion of food categories were appropriately distributed into 3 meals and snacks consumed between meals; the participants’ food preferences were considered. The adjustment of daily energy requirement based on weight loss during the study period was not performed.

#### 3) Common protocols for both groups

We conducted an open randomized clinical trial without the blinding of participants in terms of the nature of their dietary intervention. No specific meals or menus were given to the participants, and they were free to consume any food based on the recommendations provided. One registered dietitian provided nutritional education and instruction for 1 hour at week 0 and week 4, which helped participants make appropriately assigned diet plans using educational materials. The food consumption status of each participant was checked once a week by a dietitian via a telephone consultation. The dietitian reminded patients about the dietary guidelines and cooking methods that were previously described during dietary education, provided counseling to participants and answered questions, and encouraged the patients to record daily food consumption. The duration of education was similar for both groups. No additional functional foods or vitamin supplements including vitamin B12 were permitted. The participants were asked to maintain the usual level of physical activity, and to not modify their exercise habits during the intervention period.

Participants were asked to maintain their current medication, without any control of the dose or type of medication for 12 weeks; however, dose reduction was permitted when it was necessary according to a physician’s judgment. The glucose levels were measured as a part of blood sampling at baseline, and during the fourth and twelfth week; most participants self-assessed their blood glucose levels via finger-stick glucose measurement. The study protocol was reviewed and approved by the institutional review board of Kyungpook National University Hospital (IRB No: KNUH 2012-03-032). Written informed consent was obtained from all the subjects before they were enrolled in the study. All participants received a financial incentive (approximately US$ 125). This study was registered with the Clinical Research Information Service (CRiS, Korea, https://cris.nih.go.kr; registration number: KCT0001771).

### Measurement

A dietitian conducted 24-hour dietary recalls through unannounced telephone calls a total of 12 times (4 times per month, including 3 on weekdays and once on a weekend). The dietary intake of the subjects prior to participation in the trial was not assessed. Energy and nutrition intake was analyzed using a country-specific food-nutrient database (Can-Pro 4.0 professional version, the Korean Nutrition Society, Korea, 2011). Physical examination and laboratory measurements, including body weight, height, waist circumference, and blood pressure, were assessed at week 0, 4, and 12. Venous blood was collected in the morning, after fasting from 8:00 PM on the previous night. The fasting blood glucose level was determined using the hexokinase method with the ADVIA 2400 analyzer (Siemens, USA). The HbA1c level was determined with the turbidimetric inhibition immunoassay using COBAS Integra 800 (Roche, Switzerland). The levels of total cholesterol and triglyceride were analyzed using the enzymatic assay and the level of high-density lipoprotein (HDL)-cholesterol was analyzed by using the selective inhibition method with the ADVIA 2400 analyzer (Siemens, USA). The level of low-density lipoprotein (LDL)-cholesterol was estimated using the equation of total cholesterol—(triglycerides/5)—HDL cholesterol.

### Compliance based on self-assessed daily records

Each participant was required to fill out a daily dietary record form regarding the types and amount of food consumed during the intervention period. Compliance was measured according to a daily 10-point scale via self-assessed dietary recording. In the vegan diet group, the dietitian deducted 1 point whenever the consumption of meats, poultry, fish, daily goods, or eggs was entered into the daily dietary record. In the KDA diet group, the dietitian checked the types and amount of food recorded in the daily food diary and deducted 1 point whenever daily food consumption had not been maintained according to the prescribed food exchange lists. We used the average value of the daily compliance score over 0 to 12 weeks, 0 to 4 weeks, and 5 to 12 weeks.

### Statistical analysis

The primary endpoint was the HbA1c level. Repeated-measures analysis of variance (RM-ANOVA) was used to evaluate 1) whether there was a difference in HbA1c levels between the baseline and endpoint in both groups and 2) whether the effect of dietary intervention was different over time, including the interaction of the time and diet group (time*diet group). The secondary endpoints were body mass index (BMI), waist circumference, systolic/diastolic blood pressure (SBP/DBP), fasting blood glucose, LDL-cholesterol, HDL-cholesterol, and triglyceride. For intention-to-treat (ITT) analysis, we imputed the missing HbA1c levels at the fourth and twelfth weeks by using the HbA1c level at baseline by the last-observation-carried-forward imputation method. The number of missing data points were total 26 points on 13 withdrawal participants. Moreover, the clinical and dietary information required for adjustment were also imputed with the median value of each variable among the 93 subjects with complete data. All analyses were calculated using SAS 9.4 (SAS Institute Inc., Cary, NC). A p-value of <0.05 was considered statistically significant.

## Results

### Demographic and clinical characteristics at baseline

**Fig 1:**
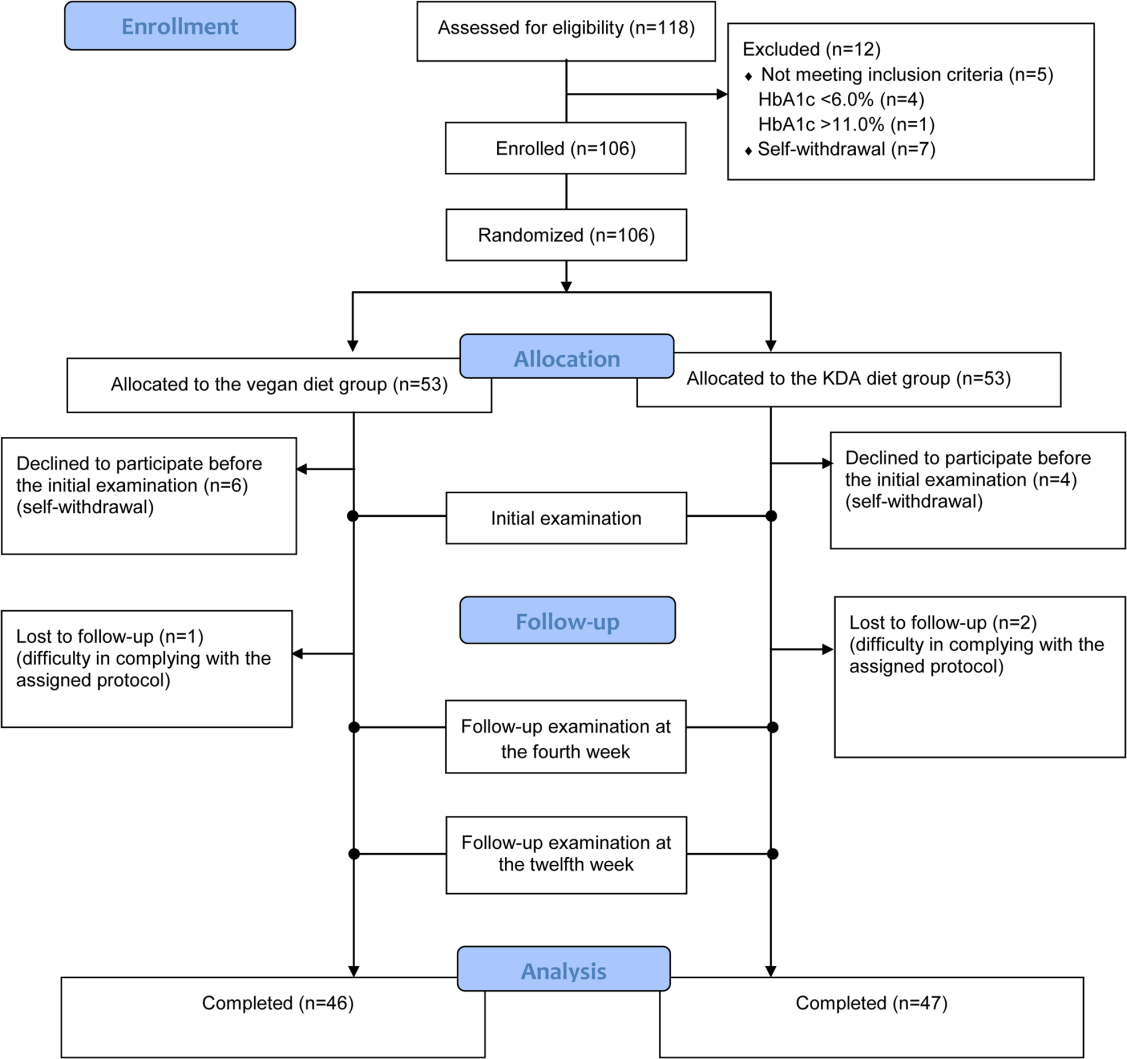
Enrollment of participants in the study.

Among the 106 randomized participants, 6 in the conventional KDA diet group and 7 in the vegan diet group dropped out of the study; hence, a total of 47 and 46 participants finally completed the 12-week intervention in the conventional KDA diet group and vegan diet group, respectively. All 13 drop-out subjects did not receive any education and did not participate in the 4-week and 12-week follow-up evaluation. Fig 1 depicts the inclusion and exclusion of the participants in the study.

When comparing the demographic and clinical characteristics at baseline between the 2 groups, none of the variables were found to be significantly different (Table 1). Moreover, none of the participants changed their medication dose over the 12 weeks.

**Table 1:** General and clinical characteristics^1^^)^ of study participants at baseline (week 0).

	Vegan diet	Conventional diet recommended by the Korean Diabetes Association	
Characteristics	n = 46	n = 47	pvalue^2^^)^
Female [n (%)]	40 (87.0)	35 (74.5)	0.128
Age (years) [range]	57.5±7.7 [32–70]	58.3±7.0 [40–69]	0.593
Duration since diabetes diagnosis (years)	9.4±8.1	9.4±5.6	0.995
Receiving insulin [n (%)]	7 (15.2)	8 (17.0)	0.813
Glargine [n (%)]	6 (13.0)	3 (6.4)	
Premixed insulin [n (%)]	0 (0.0)	2 (4.3)	
NPH^3^^)^ [n (%)]	1 (2.2)	1 (2.1)	
Glargine+rapid-acting analog [n (%)]	0 (0.0)	2 (4.3)	
Receiving metformin [n (%)]	34 (73.9)	36 (76.6)	0.764
Receiving sulfonylurea [n (%)]	17 (37.0)	21 (44.7)	0.449
Receiving other diabetes medications [n (%)]	14 (30.4)	19 (40.4)	0.314
Receiving hypertension medication [n (%)]	18 (39.1)	22 (46.8)	0.455
Receiving hypercholesterolemia medication [n (%)]	23 (50.0)	26 (55.3)	0.608
History of eye involvement [n (%)]	6 (13.0)	6 (12.8)	0.968
MNSI^4^^)^ score [median (range)]	0 [0–6]	0 [0–4]	0.555
Body mass index (kg/m^2^)	23.9±3.4	23.1±2.4	0.191
Waist circumference (cm)	85.0±9.8	82.3±7.5	0.143
Systolic blood pressure (mmHg)	125.1±16.1	128.1±19.9	0.425
Diastolic blood pressure (mmHg)	75.6±10.9	78.1±12.1	0.305
LDL-cholesterol (mg/dL)	92.7±28.5	102.8±39.0	0.155
Triglyceride (mg/dL)	130.3±61.7	147.7±113.8	0.362
HDL-cholesterol (mg/dL)	50.0±12.3	51.2±13.3	0.639
Fasting blood glucose (mg/dL)	138.4±52.4	126.3±37.7	0.205
HbA1c (%)	7.7±1.3	7.4±1.0	0.268

^1)^ percentage (%) or mean±standard deviation

^2)^calculated from the chi-square test for categorical variables or Student's t-test for continuous variables

^3)^ Neutral Protamine Hagedorn (an intermediate-acting insulin)

^4)^ Michigan Neuropathy Screening Instrument: A higher score (out of a maximum of 13 points) indicates a greater number of neuropathic symptoms.

### Nutrient intake during the intervention period

The average energy intake over the 12 weeks was 1,496 kcal/day in the vegan diet group and 1,559 kcal/day in the conventional diet group, and the difference was significant (p = 0.042; Table 2). The intake of nutrients primarily from plant-based foods (carbohydrates, vegetable fat, fiber, beta-carotene, vitamin E, vitamin K, vitamin C, vitamin B6, folate, phosphorus, and potassium) was significantly higher in the vegan diet group than in the conventional diet group. The intake of nutrients primarily from animal-based foods (animal fat, protein, cholesterol, total fatty acid, saturated fatty acid, mono-unsaturated fatty acid, vitamin D, vitamin B12, and iron) were significantly higher in the conventional diet group than in the vegan diet group. The nutrient intakes at week 1, week 4, and week 12 are described in S1 Table.

**Table 2:** Mean nutrient intake status and compliance of the study participants during the 12-week intervention period involving a vegan or conventional diet.

	Vegan diet n = 46	Conventional diet recommended by the Korean Diabetes Association n = 47	p-value^1^^)^
**Nutrients**			
Energy (kcal)	1,496.2±104.8	1,559.7±181.6	0.042
Carbohydrate (g)	268.4±19.7	249.1±35.5	0.002
Fat (g)	31.8±6.3	34.7±7.8	0.054
Animal fat (g)	2.4±1.5	14.1±5.3	<0.001
Vegetable fat (g)	29.5±6.6	20.6±5.4	<0.001
Protein (g)	55.1±5.8	66.1±9.1	<0.001
Animal protein (g)	6.4±3.7	28.3±8.2	<0.001
Plant protein (g)	48.7±5.8	37.8±6.1	<0.001
Cholesterol (g)	70.3±57.4	240.7±74.7	<0.001
Total fatty acid (g)	15.8±5.0	20.8±5.7	<0.001
SFA (g)	3.2±1.5	6.7±2.7	<0.001
MUFA (g)	5.8±2.2	8.7±3.4	<0.001
PUFA (g)	8.1±2.8	7.9±1.8	0.728
Fiber (g)	33.7±4.8	24.9±4.5	<0.001
Vitamin A (ug RE)	1,117.1±352.0	1,037.0±356.4	0.278
Beta-carotene (ug)	6,604.0±2,155.0	5,705.3±2,146.4	0.047
Vitamin D (ug)	0.6±0.5	3.4±1.7	<0.001
Vitamin E (ug)	19.6±3.9	16.1±3.1	<0.001
Vitamin K (ug)	384.0±199.4	265.2±81.8	<0.001
Vitamin C (mg)	135.1±33.2	112.2±25.9	<0.001
Vitamin B6 (mg)	2.1±0.2	1.7±0.4	<0.001
Folate (ug)	611.1±101.9	545.8±92.7	0.002
Vitamin B12 (ug)	4.1±1.8	8.5±2.7	<0.001
Calcium (mg)	567.3±116.1	540.4±105.4	0.245
Phosphorus (mg)	1,363.8±127.8	1,121.2±192.1	<0.001
Sodium (mg)	5,127.0±897.8	4,782.4±792.4	0.053
Potassium (mg)	3,583.4±492.5	3,101.4±526.6	<0.001
Magnesium (mg)	92.0±21.5	97.1±28.3	0.339
Iron (mg)	13.9±2.3	15.0±2.7	0.046
Zinc (mg)	10.2±1.2	10.3±1.4	0.869
**Compliance**			
Mean score during the 1st to 12th week	8.2±1.5	9.2±1.6	0.002
Mean score during the 1st to 4th week	8.6±1.3	9.5±1.4	0.003
Mean score during the 5th to 12th week	8.0±1.7	9.1±1.7	0.003
Proportion of high compliance [n (%)] (mean score from the 1st to 12th week ≥ 9)	14 (30.4%)	37 (78.7%)	<0.001

^1)^p-values calculated from the t-test in the case of continuous variables and the chi-square test in the case of categorical variables for between-group comparisons

SFA: saturated fatty acid; MUFA: mono-unsaturated fatty acid; PUFA: poly-unsaturated fatty acid

### Compliance based on the self-assessed daily records

Compliance—evaluated based on the self-assessed dietary record—was better in the conventional diet group than in the vegan diet group (Table 2). The mean compliance score (a maximum of 10 points) during the overall intervention period was 9.2±1.6 and 8.2±1.5 in the conventional diet group and vegan diet group, respectively (p = 0.002). Compared to the early phase of the trial (first to fourth week), the compliance was lower during the late phase of the trial (fifth to twelfth week) in both groups. The proportion of participants with high compliance (≥9 points) was significantly higher in the conventional diet group (78.7%) than in the vegan group (30.4%) (p<0.001).

### Primary endpoint: Glycemic control

**Fig 2:**
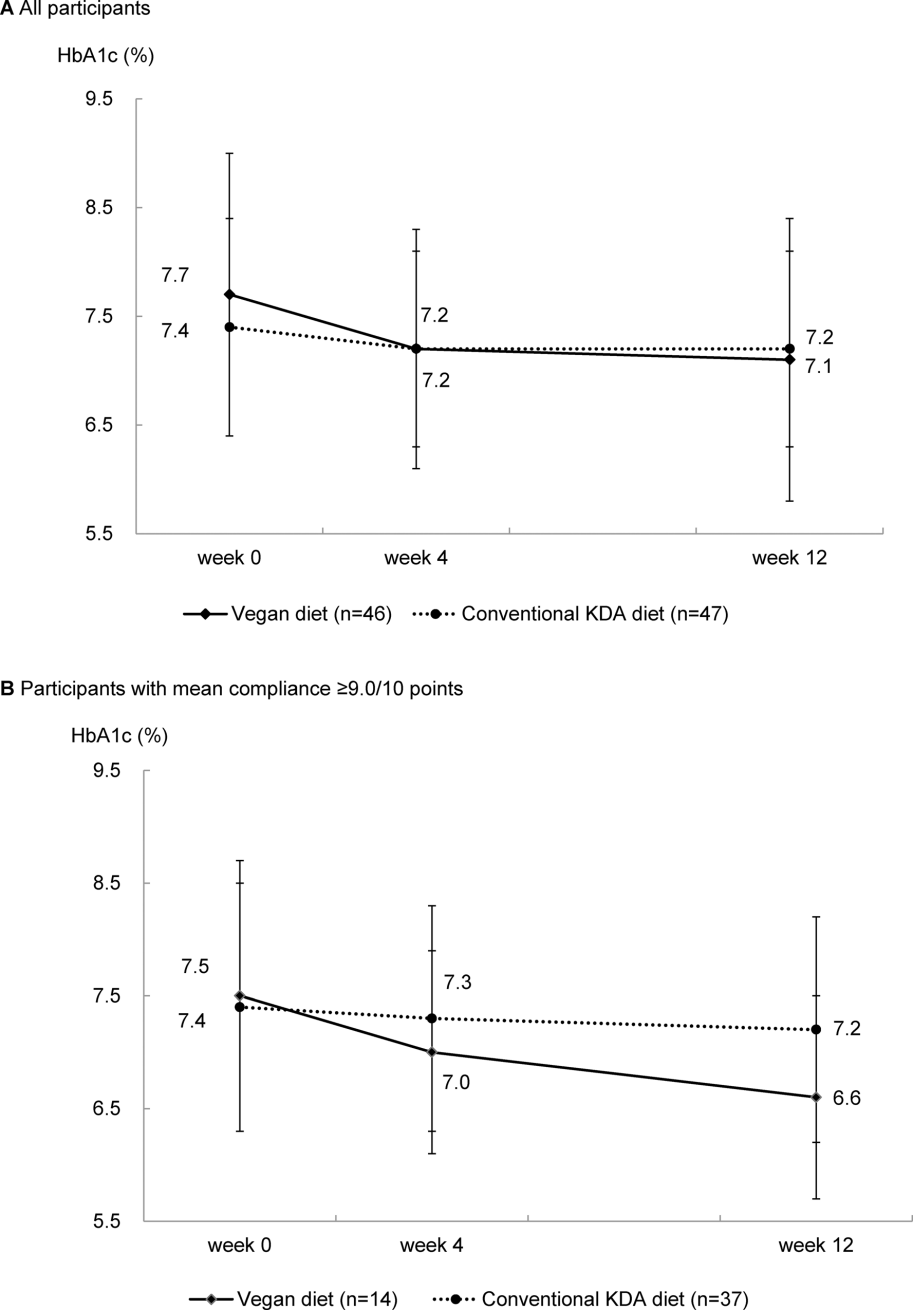
Levels of HbA1c (mean ± SD) at baseline and at 4 and 12 weeks in individuals with type 2 diabetes following a vegan or conventional diet recommended by the Korean Diabetes Association. (A) All participants. (B) Participants with mean compliance ≥9.0/10 points.

The HbA1c level significantly decreased over time in both groups: -0.5% in the vegan diet group (p<0.01) and -0.2% in the conventional diet group (p<0.05) (Table 3, Fig 2A). However, the changes in the HbA1c level from baseline to the end point were greater in the vegan diet group than in the conventional diet group (p-for-interaction for time*group interaction = 0.017). When analyses were restricted to subjects with a high compliance (≥9 points/10 points), the difference in HbA1c level changes between the 2 groups became more remarkable: -0.9% in the vegan group and -0.3% in the conventional group (p-for-interaction for time*group interaction = 0.010) (Table 3, Fig 2B). The differences remained significant even after adjusting for energy intake or waist circumference. The results of the ITT analysis were similar to those of subjects with complete data (S2 Table).

**Table 3:** Clinical outcomes of the study participants following a vegan or conventional diet at baseline, and after the fourth and twelfth weeks.

	Vegan diet				Conventional diet recommended by the Korean Diabetes Association				p value for group*time interaction^1^^)^
	n = 46				n = 47				
Clinical outcome	Week 0 (baseline)	Week 4	Week 12 (final)	Change (Week 12-Week 0)	Week 0 (baseline)	Week 4	Week 12 (final)	Change (Week 12-Week 0)	
**Primary endpoint**									
HbA1c (%) (all participants)	7.7±1.3	7.2±1.1	7.1±1.3	-0.5±0.8^‡^	7.4±1.0	7.2±0.9	7.2±0.9	-0.2±0.7^†^	0.017 (0.037)^2^^)^ (0.042)^3^^)^
HbA1c (%) [compliance≥9.0 (n = 14 in vegan, n = 37 in KDA)]	7.5±1.2	7.0±0.9	6.6±0.9	-0.9±0.8^‡^	7.4±1.1	7.3±1.0	7.2±1.0	-0.3±0.7^†^	0.010 (0.013)^2^^)^ (0.011)^3^^)^
**Secondary endpoint**									
BMI (kg/m^2^)	23.9±3.4	23.8±3.4	23.5±3.4	-0.5±0.9^‡^	23.1±2.4	23.1±2.3	23.0±2.4	-0.1±0.6	0.092
Waist circumference (cm)	85.0±9.8	82.8±9.7	81.9±9.9	-3.1±4.9^‡^	82.3±7.5	82.1±7.6	81.5±7.9	-0.8±4.6	0.027
Systolic BP (mmHg)	125.1±16.1	124.4±16.1	126.1±14.4	1.0±14.9	128.1±19.9	121.9±16.5	126.6±16.1	-1.5±18.7	0.186
Diastolic BP (mmHg)	75.6±10.9	74.5±10.5	76.7±9.3	1.1±9.0	78.1±12.1	75.2±10.1	76.7±10.3	-1.4±9.9	0.335
Fasting blood glucose (mg/dL)	138.4±52.4	117.3±32.1	125.2±38.0	-13.2±47.4	126.3±37.7	119.7±32.7	126.3±33.0	0.0±39.1	0.146
LDL-cholesterol (mg/dL)	92.7±28.5	89.1±31.2	89.9±32.3	-2.8±17.8	102.8±39.0	97.8±36.1	101.9±38.5	-1.0±29.3	0.732
Triglyceride (mg/dL)	130.3±61.7	128.7±60.3	143.7±92.4	13.4±72.8	147.7±113.8	141.9±91.9	128.8±57.9	-18.9±81.9	0.053
HDL-cholesterol (mg/dL)	50.0±12.3	49.5±11.9	52.2±14.9	2.2±8.8	51.2±13.3	50.8±13.1	51.7±13.0	0.5±8.2	0.459

^1)^ p values for the group*time interaction were calculated via repeated measures analysis of variance or MANOVA (Wilks' lambda)

^2)^ p values for the group*time interaction after adjusting for the mean energy intake (kcal) over the 12-week period

^3)^ p values for the group*time interaction after adjusting for waist circumference at 0, 4, and 12 weeks

^†^p<0.05

^‡^p<0.01; p values represent the values of the paired t-test that assessed whether the changes from baseline to the final week were significantly different from zero.

None of the participants required any change in medication during the intervention period

### Secondary endpoint: BMI, waist circumference, blood pressure, and lipid control

The BMI and waist circumference significantly reduced over the 12-week period only in the vegan diet group (p-for-interaction for time*group interaction = 0.027 for waist circumference). However, there were no significant differences in the changes in SBP, DBP, LDL-cholesterol level, and HDL-cholesterol level in both the groups. The triglyceride levels tended to increase in the vegan diet group and tended to decrease in the conventional diet group (p-for-interaction for time*group interaction = 0.053). When the analyses were restricted to subjects with a high compliance (≥9 points/10 points), the results remained unchanged (data not shown).

## Discussion

In the present study, we observed that both vegan and conventional diabetic diets were significantly associated with reductions in HbA1c levels. However, compared to the conventional diet, the vegan diet appeared to be more effective for glycemic control among T2D patients. In particular, the vegan diet group with a high compliance showed a markedly decreasing trend in the HbA1c level.

Importantly, the benefit of the vegan diet was noted even after adjusting for energy intake and waist circumference over the 12-week period between the 2 groups. At present, weight loss due to reduced total calorie intake is considered to be the main mechanism for achieving good glycemic control in T2D patients with various diet interventions, including a vegan or vegetarian diet [12]. However, the current study suggests that a vegan diet may have additional benefits, other than the low energy intake or weight loss.

Consistent with the findings of the current study, a recent meta-analysis including 6 controlled clinical trials conducted primarily in the United States showed that a vegetarian or vegan diet has a significant glycemic control effect in the management of T2D [5]. In the pooled analysis, vegetarian diets were associated with a significant decrease in HbA1c levels (-0.39%), as compared to omnivorous diets. This difference appears to be slightly larger than that observed in the overall sample in the present study; in fact, the difference in the changes in HbA1C levels between the vegan diet group (-0.5%) and conventional diet group (-0.2%) was approximately -0.3%.

These findings appear to fail to support our priori hypothesis that a vegan diet would be more effective in T2D management among Asians than among Westerners when the absolute value of decreased HbA1c between studies was simply compared. However, when we only compared participants with good compliance, we noted that the HbA1C reduction among the vegan diet group was 0.9%, and that the difference between the 2 groups was doubled (-0.6%). As previous clinical trials did not report results only from participants with good compliance, it is still difficult to interpret this finding as direct evidence for the greater effectiveness of a vegan diet in T2D management among Asians than among Westerners.

Nevertheless, the reduction of HbA1C levels observed in the vegan group with good compliance appeared to be larger than the effects of other dietary approaches, which were examined in a meta-analysis of 20 RCTs on various T2D diet interventions; the largest effect was observed with a Mediterranean diet (effect size, -0.47%) [12]. In RCTs, low compliance can be expected with vegan diets as it would be difficult for individuals with a prior omnivorous diet to maintain a vegan diet for several months. However, our findings suggest that, if patients are motivated to maintain a vegan diet, it can be effective in the management of T2D.

The practical advantages of vegan diets include the absence of any restriction on calorie intake, lack of necessity for calculating food portion sizes, and ease of understanding the diet methods (no consumption of animal food) [13]. In particular, compared to conventional diabetic diets that are focused on restricting calorie intake and portion size, vegetarian diets are reportedly easier to follow during exercise—another key component of T2D management—due to the reduced feeling of hunger [14]. Considering that both diet and exercise are 2 vital components for the optimal management of T2D, such advantages would be important for T2D patients.

In addition to improved glycemic control, vegetarian or vegan diets may offer health benefits associated with cardiovascular risk factors such as serum lipids and blood pressure, as compared to omnivorous diets [15]. In a meta-analysis on 11 RCTs, vegetarian or vegan diets were found to have significant lowering effects on the concentrations of LDL-cholesterol and HDL-cholesterol, but no remarkable effect on the concentration of triglycerides [16]. Moreover, in a meta-analysis on 7 RCTs, these diets were found to be effective in reducing SBP and DBP, in comparison with omnivorous diets [17]. However, we did not observe any benefit on blood pressure and lipids in both diets. The effect of a vegan diet on the cardiovascular risk factors may be better evaluated among participants with dyslipidemia or hypertension than among those with T2D.

Similar to other T2D diet interventions, weight loss (particularly the loss of visceral fat), as a result of lower energy intake, has been considered a main mechanism for improved glycemic control with vegan or vegetarian diets [13, 14, 18]. However, the benefit of vegan diets in the present study could not be fully explained by weight loss or lower energy intake. Other potential mechanisms may include the higher intake of dietary fiber [19]. Dietary fiber can lower the glycemic index of carbohydrates by slowing the absorption of glucose from the intestine [20]. In addition, dietary fiber can improve glycemic control by increasing bile acid excretion [21], and increasing the production of short-chain fatty acids via the bacterial fermentation of fiber [22]. The reduction of intramyocellular lipid concentrations may represent another possible mechanism [23].

Another explanation may involve the reduced exposure to POPs by vegan diets through the prohibition of animal food. Recently, background exposure to low-dose POPs has been found to be an important risk factor of developing T2D [8]. Furthermore, these chemicals are associated with poor glycemic control among T2D patients [24], and with an increased risk of cardiovascular diseases [25, 26], which are the most common complications of T2D. Hence, avoiding animal food contaminated with POPs may offer additional benefit for T2D patients.

There are several limitations of the present study. First, the study duration of 3 months would not be sufficient to evaluate the long-term effects of vegan diets on glycemic control. However, an RCT with a long duration would commonly involve changes in the medication for appropriate medical management, and hence, the estimation of effect size might be complicated [6]. Hence, a 3-month study duration without any change in medication would be suitable for the evaluation of diet effects among T2D patients. Second, compared to previous trials on diet intervention that used a more intensive approach (including weekly or biweekly group meetings), this study used only telephone consultations. This may be the reason for the low compliance among our study subjects. Third, most of the study participants were women, as men were difficult to recruit for diet intervention. In fact, it is known that the participants of clinical trials on interventions for diet or behavioral change are predominantly female due to the low participation rate of males [27]. Nevertheless, we could not exclude the possibility that the effect of vegan diets may differ according to the gender. Fourth, it is unclear whether the improvement in glycemic control through vegan diets would lead to a reduction in the risks of macro- or microvascular complications of diabetes, as there are certain concerns regarding the potential of low intake of some nutrients in a vegan diet that is not planned well [28].

## Conclusion

The use of a vegan diet for 3 months was found to be more effective for glycemic control among T2D patients, as compared to a conventional diabetes diet recommended by the KDA. However, as the compliance of the vegan diet group was lower than that of the conventional group, and because dietary choices are often personal, it is not realistic to recommend vegan diets to all T2D patients. Nevertheless, this effective diet approach can be applied for T2D patients who are strongly motivated to follow a vegan diet, particularly in the Asian population.

## Supporting Information

S1 CONSORT Checklist (DOC)

S1 Protocol (DOC)

S1 Table Nutrient intake status of the study participants following a vegan or conventional diet after the first, fourth, and twelfth weeks. (DOCX)

S2 Table Primary endpoint (HbA1c) among all randomized participants using an intention-to-treat analysis (DOCX)

S1 Text Trial protocol (Korean). (DOC)
