# Supplementary material for: Effect of a Brown Rice Based Vegan Diet and Conventional Diabetic Diet on Glycemic Control of Patients with Type 2 Diabetes: A 12-Week Randomized Clinical Trial
Source: PLoS One. 2016 Jun 2;11(6):e0155918. doi: 10.1371/journal.pone.0155918 (PMC4890770; doi:10.1371/journal.pone.0155918)
Supplement: S1 Protocol — (DOC) [file pone.0155918.s002.doc]

Trial protocol
Title	Evaluation of clinical effects and POPs-related mechanisms of vegan diet among Patients with Type 2 Diabetes 	
Institution	Department of Preventive Medicine, School of Medicine, Kyungpook National University
· Address: 
680 Gukchaebosang-ro, Jung-gu, Daegu, 41944, Korea	
Principal Investigator
(Contact information) 	Duk-Hee Lee, M.D., PhD
Department of Preventive Medicine
School of Medicine
Kyungpook National University
fax: +82-53-425-2447
e-mail: lee_dh@knu.ac.kr	
Funding	Ministry of Health and Welfare (Republic of Korea)	
Background & Objective	Recent some studies have suggested that vegetarian and vegan diet have health benefits for a control and management of type 2 diabetes. We tried to evaluate a glycemic control effects of vegan diet and conventionally recommended diabetic diet by using the randomized clinical trial. Participants with type 2 diabetes will be randomly assigned to a vegan diet group or a conventional diet group recommended by the Korean Diabetes Association 2011 for 12 weeks.	
Study duration	2012-04-03 ~ 2012-10-31	
Study design	· type: prospective & interventional
· intervention model: parallel
· blinding/masking: open
· allocation methods/allocation ratio: randomized / 1:1	
Randomization 	· type: stratified block randomization
· strata: 2 (HbA1c <8.0% and ¡Ã8.0%)
· group: 2 (A–control group, B-experimental group)
· After a generation of the random blocks of allocation (block size=4) using proc plan procedure by the SAS 9.2 software program, a principal investigator write the random blocks (the allocated group) on paper and seal an envelope. A researcher placed the envelopes and assigned the list of participants according to the enrollment order.	
Experimental group	vegan diet consisted of whole grains, vegetables, fruit, and legumes for 12 weeks	
Control group	conventional diet followed treatment guidelines for diabetes recommended by Korean Diabetic Association (KDA) 2011 for 12 weeks	
Subject Eligibility	
previously diagnosed with type 2 diabetes 
	
Sample size	106 (Experimental: 53, Control: 53)
The sample size was calculated as 53 in each group based on the previous study using the formula; assuming a between-group HbA1c difference (effect size) of 0.65%p, standard deviation of 1.0, ¥á level of 0.05, power of 80%, d a loss of follow-up rate of 30%. 

: change of a outcome variable levels during 12 weeks in control group 
: change of a outcome variable levels during 12 weeks in experimental group 
: variance a outcome variable	
Inclusion criteria	· previously diagnosed with type 2 diabetes 
· age: from 30 to 70 years old
· use of hypoglycemic medications for ¡Ã 6 months 
· 6.5% ¡Â glycosylated hemoglobin (HbA1c) ¡Â 11.0%  	
Exclusion criteria	· increased dose of hypoglycemic medication or addition a new drug during recent 2 months 
· smoker or drinker 
· current vegetarian 
· pregnancy 
· severe complications (e.g.,chronic renal failure)  	
Stopping criteria	¡¤ when a doctor determines that there is a potential to do harm if a participant continues the trial	
Outcome measure	1) Primary outcome: change of HbA1c levels during 12 weeks
2) Secondary outcome: change of BMI, waist circumference, triglyceride levels, LDL-cholesterol, HDL-cholesterol, fasting blood glucose level, systolic/diastolic blood pressure 	
statistical methods	To compare the change of outcome variable levels during 12 weeks in two groups by the time, the repeated measured ANOVA will be used 	
References	1. American Diabetes Association. Nutrition Recommendations and Interventions for Diabetes. A position statement of the American Diabetes Association. Dibetes Care, Volume 31, Supplement 1, January 2008
2. Barnard ND, Scialli AR, Turner-McGrievy G, Lanou AJ, Glass J. The effects of a low-fat, plant-based dietary intervention on body weight, metabolism, and insulin sensitivity. Am J Med. 2005;118:991–.997
3. Turner-McGrievy GM, Barnard ND, Scialli AR. A two-year randomized weight loss trial comparing a vegan diet to a more moderate low-fat diet. Obesity (Silver Spring). 2007;15:2276–. 2281.
4. Barnard ND, Cohen J, Jenkins DJ, et al. A low-fat vegan diet improves glycemic control and cardiovascular risk factors in a randomized clinical trial in individuals with type 2 diabetes. Diabetes Care. 2006;29:1777–.1783.
5. Neal D Barnard, Joshua Cohen, David JA Jenkins, Gabrielle Turner-McGrievy, Lise Gloede, Amber Green, and Hope Ferdowsian.A low-fat vegan diet and a conventional diabetes diet in the treatment of type 2 diabetes: a randomized, controlled, 74-wk clinical trial. Am J Clin Nutr 2009;89(suppl):1588S–-96S 	
