# Supplementary material for: Effect of a Brown Rice Based Vegan Diet and Conventional Diabetic Diet on Glycemic Control of Patients with Type 2 Diabetes: A 12-Week Randomized Clinical Trial
Source: PLoS One. 2016 Jun 2;11(6):e0155918. doi: 10.1371/journal.pone.0155918 (PMC4890770; doi:10.1371/journal.pone.0155918)
Supplement: S1 Table — (DOCX) [file pone.0155918.s003.docx]

S1 Table. Nutrient intake status of the study participants following a vegan or conventional diet at the first, fourth, and twelfth week

|  | Vegan diet | | |  | Conventional diet recommended by the Korean Diabetes Association | | |  | p  value^1)^ |
| --- | --- | --- | --- | --- | --- | --- | --- | --- | --- |
|  | n=46 | | |  | n=47 | | |  |  |
| Nutrients | Week 1 | Week 4 | Week 12 | Change  (Week 12-Week 0) | Week 1 | Week 4 | Week12 | Change  (Week 12-Week 0) |  |
| Energy (kcal) | 1569.9±211.8 | 1518.2±229.4 | 1498.6±204.8 | -71.3±280.9 | 1599.7±268.8 | 1623.5±337.9 | 1532.3±262.8 | -67.4±301.2 | 0.949 |
| Carbohydrate (g) | 280.9±44.1 | 262.9±36.2 | 269.0±41.0 | -11.9±54.0 | 245.8±51.1 | 250.1±54.5 | 249.1±49.1 | 3.4±44.3 | 0.140 |
| Fat (g) | 34.8±11.4 | 35.6±15.3 | 32.0±12.7 | -2.8±17.2 | 38.0±15.4 | 38.0±18.4 | 32.8±13.5 | -5.3±19.6 | 0.527 |
| Animal fat (g) | 1.4±1.6 | 4.2±8.7 | 2.9±5.6 | 1.5±6.0 | 15.8±11.7 | 17.0±18.2 | 12.1±10.4 | -3.8±14.5 | 0.025 |
| Vegetable fat (g) | 33.4±11.6 | 31.4±13.4 | 29.1±12.3 | -4.3±16.9 | 22.2±12.2 | 21.1±10.4 | 20.7±9.9 | -1.5±15.8 | 0.406 |
| Protein (g) | 56.6±11.3 | 57.9±14.4 | 54.0±10.0 | -2.6±14.1 | 72.1±20.2 | 71.6±27.6 | 61.9±16.4 | -10.1±27.5 | 0.101 |
| Animal protein (g) | 3.8±4.1 | 9.5±14.4 | 6.8±6.6 | 3.0±8.4 | 33.1±19.5 | 33.3±29.0 | 25.2±17.6 | -7.8±25.7 | 0.008 |
| Plant protein (g) | 52.8±11.0 | 48.4±11.2 | 47.2±9.4 | -5.6±13.3 | 39.0±9.8 | 38.4±11.5 | 36.7±9.3 | -2.3±12.3 | 0.213 |
| Cholesterol (g) | 47.2±67.4 | 79.9±101.8 | 75.2±89.7 | 28.0±110.1 | 241.3±166.1 | 256.9±201.3 | 249.0±206.0 | 7.7±232.7 | 0.592 |
| Total fatty acid (g) | 17.8±8.2 | 17.8±12.9 | 15.6±8.6 | -2.1±10.6 | 24.1±14.3 | 21.5±15.3 | 19.2±10.6 | -4.9±18.2 | 0.377 |
| SFA (g) | 2.9±1.4 | 3.2±3.0 | 2.6±1.5 | -0.3±1.8 | 8.9±10.3 | 6.0±6.3 | 7.5±11.9 | -1.4±15.8 | 0.634 |
| MUFA (g) | 5.8±3.2 | 6.1±4.4 | 5.0±2.9 | -0.8±4.1 | 11.6±13.5 | 7.8±6.7 | 10.3±15.8 | -1.3±21.1 | 0.866 |
| PUFA (g) | 9.1±4.2 | 8.5±6.0 | 8.0±4.6 | -1.1±5.3 | 9.0±5.4 | 7.7±4.3 | 8.2±5.4 | -0.8±7.9 | 0.842 |
| Fiber(g) | 36.6±8.2 | 33.7±9.1 | 34.3±7.5 | -2.3±9.4 | 26.8±9.3 | 25.1±8.1 | 23.8±6.4 | -2.9±10.5 | 0.755 |
| Vitamin A (ug RE) | 1290.8±603.2 | 1100.5±571.7 | 1022.5±613.5 | -268.3±735.1 | 1199.0±681.6 | 1111.6±673.5 | 794.6±455.8 | -404.4±766.3 | 0.385 |
| Beta-carotene (ug) | 7695.9±3637.5 | 6473.4±3470.8 | 6006.1±3669.2 | -1,689.8±4,367.5 | 6697.5±4147.6 | 6143.7±4111.6 | 4330.2±2610.9 | -2,367.3±4,498.4 | 0.463 |
| Vitamin D (ug) | 0.3±0.4 | 0.7±1.7 | 0.5±0.8 | 0.2±1.0 | 4.1±6.9 | 3.0±4.3 | 1.7±2.4 | -2.4±7.2 | 0.019 |
| Vitamin E (ug) | 21.8±5.7 | 21.1±8.3 | 20.8±8.6 | -0.9±10.0 | 16.5±6.7 | 16.8±8.2 | 15.4±6.8 | -1.1±9.1 | 0.940 |
| Vitamin K (ug) | 376.9±258.7 | 464.9±389.9 | 305.7±216.5 | -71.2±299.8 | 315.6±217.5 | 282.9±193.5 | 198.6±104.9 | -117.0±224.3 | 0.405 |
| Vitamin C (mg) | 163.8±77.1 | 136.8±63.7 | 128.2±52.8 | -35.5±77.0 | 122.7±55.2 | 109.5±48.1 | 99.1±38.5 | -23.5±59.9 | 0.403 |
| Vitamin B6 (mg) | 2.3±0.4 | 2.1±0.4 | 2.1±0.4 | -0.2±0.5 | 1.8±0.6 | 1.7±0.6 | 1.6±0.5 | -0.2±0.7 | 0.633 |
| Folate (ug) | 690.6±258.9 | 663.2±198.9 | 593.5±151.4 | -97.1±291.9 | 593.3±201.2 | 563.9±183.0 | 489.1±139.9 | -104.1±210.1 | 0.895 |
| Vitamin B12 (ug) | 3.4±2.6 | 5.1±7.5 | 4.2±3.3 | 0.8±4.3 | 9.9±9.8 | 8.8±6.8 | 7.4±5.8 | -2.4±12.4 | 0.095 |
| Calcium (mg) | 576.0±197.7 | 590.0±225.7 | 548.2±188.7 | -27.9±271.3 | 629.0±285.2 | 514.0±211.8 | 471.3±213.9 | -157.7±356.8 | 0.052 |
| Phosphorus (mg) | 1451.0±228.2 | 1373.1±259.0 | 1332.0±250.9 | -119.0±292.6 | 1204.8±315.7 | 1154.9±338.4 | 1054.4±288.2 | -150.4±399.2 | 0.666 |
| Sodium (mg) | 5062.7±1610.0 | 5399.4±1620.8 | 5215.1±1555.9 | 152.3±1,972.3 | 4971.5±1682.2 | 4553.8±1649.3 | 4625.5±1310.9 | -346.0±1,781.7 | 0.204 |
| Potassium (mg) | 4086.1±1136.9 | 3646.8±805.7 | 3594.9±1029.4 | -491.2±1,397.3 | 3275.4±884.5 | 3201.2±848.8 | 2894.2±720.8 | -381.1±961.0 | 0.660 |
| Magnesium (mg) | 108.2±68.9 | 90.8±49.7 | 96.5±60.3 | -11.7±95.6 | 90.2±51.3 | 104.4±54.0 | 94.9±54.8 | 4.8±70.6 | 0.349 |
| Iron (mg) | 14.6±4.0 | 14.7±3.8 | 13.5±3.8 | -1.1±5.0 | 15.8±4.8 | 15.5±4.7 | 14.2±4.2 | -1.6±5.4 | 0.636 |
| Zinc (mg) | 11.1±2.2 | 10.6±2.1 | 9.9±2.1 | -1.1±2.5 | 10.8±2.9 | 11.0±3.1 | 9.6±2.2 | -1.2±3.3 | 0.885 |

^1)^ p values calculated from the t-test for between-group comparisons of changes from week 0 to week 12

SFA: saturated fatty acid

MUFA: mono-unsaturated fatty acid

PUFA: poly-unsaturated fatty acid
