# Supplementary material for: Effect of a Brown Rice Based Vegan Diet and Conventional Diabetic Diet on Glycemic Control of Patients with Type 2 Diabetes: A 12-Week Randomized Clinical Trial
Source: PLoS One. 2016 Jun 2;11(6):e0155918. doi: 10.1371/journal.pone.0155918 (PMC4890770; doi:10.1371/journal.pone.0155918)
Supplement: S2 Table — (DOCX) [file pone.0155918.s004.docx]

S2 Table. Primary endpoint (HbA1c) among all randomized participants using intention-to-treat analysis

|  | Vegan diet | | |  | Conventional diet recommended by the Korean Diabetes Association | | |  | p-  value for group*time interaction^1)^ |
| --- | --- | --- | --- | --- | --- | --- | --- | --- | --- |
|  | n=53 | | |  | n=53 | | |  |  |
| Clinical outcome | Week 0  (baseline) | Week 4 | Week 12  (final) | Change  (Week12-Week0) | Week 0  (baseline) | Week4 | Week 12  (final) | Change  (Week12-Week0) |  |
| Primary endpoint |  |  |  |  |  |  |  |  |  |
| HbA1c (%) | 7.7±1.3 | 7.3±1.1 | 7.2±1.2 | -0.5±0.8^‡^ | 7.5±1.1 | 7.3±1.0 | 7.3±1.1 | -0.2±0.7^†^ | 0.026  (0.054)^2)^  (0.058)^3)^ |

^1)^ p values for the group*time interaction were calculated via repeated measures analysis of variance or MANOVA (Wilks' lambda)

^2)^ p values for the group*time interaction after adjusting for the mean energy intake (kcal) over the 12-week period

^3)^ p values for the group*time interaction after adjusting for waist circumference at 0, 4, and 12 weeks

^†^p<0.05, ^‡^p<0.01; p values represent the values of the paired t-test that assessed whether the changes from baseline to the final week were significantly different from zero.
