# Supplementary material for: Effect of a Brown Rice Based Vegan Diet and Conventional Diabetic Diet on Glycemic Control of Patients with Type 2 Diabetes: A 12-Week Randomized Clinical Trial
Source: PLoS One. 2016 Jun 2;11(6):e0155918. doi: 10.1371/journal.pone.0155918 (PMC4890770; doi:10.1371/journal.pone.0155918)
Supplement: S1 Text — (DOC) [file pone.0155918.s005.doc]

¿¬±¸ °èÈ¹¼­

1. ¿¬±¸ÀÇ ¸íÄª ¹× ´Ü°è
- ¸íÄª : Á¦ 2Çü ´ç´¢º´¿¡¼­ Ã¤½ÄÀÇ ÀÓ»óÈ¿°ú Æò°¡ ¹× POPs °ü·Ã ±âÀü ¿¬±¸
- ´Ü°è : ÇÐ¼ú¿ë

2. ¿¬±¸ÀÇ ½Ç½Ã±â°ü, ÀÓ»ó½ÃÇè Ã¥ÀÓÀÚ ¹× °øµ¿¿¬±¸¿ø
- È¸»ç(½Ã°ü) : °æºÏ´ëÇÐ±³ º´¿ø
  ÁÖ¼Ò : ´ë±¸±¤¿ª½Ã Áß±¸ »ï´ö2°¡ 50¹øÁö
- Ã¥ÀÓ ¿¬±¸ÀÚ(¼Ò¼Ó°ú/Á÷À§/¼º¸í):
  °æºÏ´ëÇÐ±³ ÀÇÇÐÀü¹®´ëÇÐ¿ø ¿¹¹æÀÇÇÐ±³½Ç / ±³¼ö / ÀÌ´öÈñ
- °øµ¿ ¿¬±¸ÀÚ(¼Ò¼Ó°ú/Á÷À§/¼º¸í):
  °æºÏ´ëÇÐ±³ º´¿ø ³»ºÐºñ³»°ú / ±³¼ö / ÀÌÀÎ±Ô
  °æºÏ´ëÇÐ±³ º´¿ø ³»ºÐºñ³»°ú / ±³¼ö / ±èÁ¤±¹

3. ¿¬±¸¹è°æ ¹× ¸ñÀû

- ¿¬±¸ ¹è°æ : Á¦ 2Çü ´ç´¢º´ÀÇ °ü¸®, ÇÕº´ÁõÀÇ ¿¹¹æ ¹× Áö¿¬¿¡ ÀÖ¾î¼­ ½ÄÀÌ¿ä¹ýÀÇ Áß¿ä¼ºÀº ¸Å¿ì Àß ¾Ë·ÁÁ® ÀÖ´Ù. ¹Ì±¹ ´ç´¢º´ ÇÐÈ¸(American Diabetic Association, ADA)¿¡¼­ ±ÇÀåÇÏ´Â ½ÄÀÌ¿ä¹ý¿¡ ±Ù°ÅÇÏ¿© ¸¸µé¾îÁø ´ëÇÑ ´ç´¢º´ ÇÐÈ¸ÀÇ ´ç´¢º´ °ü¸®¸¦ À§ÇÑ ½ÄÀÌ¿ä¹ýÀ¸·Î Åº¼öÈ­¹° ¼·Ãë ¹× Æ÷È­ Áö¹æ°ú Æ®·£½º Áö¹æ, ÄÝ·¹½ºÅ×·ÑÀÇ ¼·Ãë Á¦ÇÑ, ±×¸®°í °úÃ¼Áß È¯ÀÚ¿¡¼­ÀÇ ¿­·® ¼·Ãë Á¦ÇÑÀ» ±Ç°íÇÏ°í ÀÖÀ¸¸ç, ÇöÀç ´ëºÎºÐÀÇ º´¿ø¿¡¼­ ÀÌ¿Í ÇÔ²² °³ÀÎÀÇ ÀÇÇÐÀû »óÅÂ¿Í »ýÈ°¹æ½Ä, À½½Ä ±âÈ£µµ¿¡ ¸ÂÃá ½ÄÀÌ ¿ä¹ýÀ» ´ç´¢º´ È¯ÀÚ¿¡°Ô ±Ç°íÇÏ°í ÀÖ´Ù(1). ÇÏÁö¸¸, ÃÖ±Ù ±¹¿Ü¿¡¼­ ½ÃÇàµÈ ¸î¸î ¹«ÀÛÀ§ÀÓ»ó½ÃÇè¿¬±¸¿¡¼­ ¿­·®À» Á¦ÇÑÇÏÁö ¾Ê°í µ¿¹°¼º ½ÄÇ°À» ¸ÔÁö ¾Êµµ·Ï ÇÏ´Â Ã¤½ÄÀ» ÇÑ ±ºÀÌ ±âÁ¸ÀÇ ¹Ì±¹ ´ç´¢º´ÇÐÈ¸¿¡¼­ ±ÇÀåÇÏ´Â ´ç´¢º´ ½ÄÀÌ ¿ä¹ý±º¿¡ ºñÇØ Ç÷´ç°ü¸®¿¡ ÀÖ¾î ´õ ¿ì¼öÇÏ´Ù´Â ¿¬±¸°á°úµéÀÌ º¸°íµÇ°í ÀÖ´Ù(2-5)

- ¿¬±¸ ¸ñÀû : Á¦ 2Çü ´ç´¢º´À» Áø´Ü¹Þ°í Ä¡·áÁßÀÎ ¿©¼º È¯ÀÚµéÀ» ´ë»óÀ¸·Î 12ÁÖ°£ Çö¹ÌÃ¤½Ä ¹× ´ëÇÑ ´ç´¢º´ ÇÐÈ¸ ±Ç°í ´ç´¢º´ ½ÄÀÌ¸¦ ½ÃÇàÇÑ ÈÄ Çö¹ÌÃ¤½Ä°ú ´ç´¢º´ ½ÄÀÌ°¡ HbA1c ¹× °Ç°­ ÁöÇ¥ º¯È­¿¡ ¹ÌÄ¡´Â ¿µÇâÀ» ¾Ë¾Æº¸°í, Çö¹ÌÃ¤½Ä°ú ´ç´¢º´ ½ÄÀÌÀÇ È¿°ú¸¦ ºñ±³ÇØº¸°íÀÚ ÇÑ´Ù.


4. ÀÓ»ó½ÃÇè ½ÄÀÌ¿ä¹ý
- ½ÃÇè ½ÄÀÌ¿ä¹ý : Çö¹Ì Ã¤½Ä ½ÄÀÌ¿ä¹ý
- ´ëÁ¶ ½ÄÀÌ¿ä¹ý : ´ëÇÑ ´ç´¢º´ ÇÐÈ¸ ±Ç°í ´ç´¢º´ ½ÄÀÌ¿ä¹ý

5. ´ë»ó ÁúÈ¯ ¹× ÀÓ»ó½ÃÇè ±â°£
- ´ë»óÁúÈ¯ : Á¦ 2Çü ´ç´¢º´
- ÀÓ»ó½ÃÇè±â°£ : 2012³â 4¿ù 3ÀÏºÎÅÍ 2012³â 10¿ù 31ÀÏ±îÁö
 ․ È¯ÀÚ¸ðÁý : 2012³â 4¿ù 3ÀÏºÎÅÍ 2012³â 7¿ù±îÁö µî·ÏÇÒ ¿¹Á¤
 ․ ÃßÀû°üÂû : 2012³â 8¿ùºÎÅÍ 3°³¿ù°£

6. ¿¬±¸ ´ë»óÀÚÀÇ ¼±Á¤±âÁØ, Á¦¿Ü±âÁØ ¹× ¸ñÇ¥ ¿¬±¸ ´ë»óÀÚ ¼ö¿Í »êÁ¤±Ù°Å 

- º» ¿¬±¸ÀÇ Æ÷ÇÔ ±âÁØ
¡¤ ¸¸ 30¼¼ ÀÌ»ó 70¼¼ ÀÌÇÏ 
¡¤ »çÀü¿¡ Á¦ 2Çü ´ç´¢º´À» Áø´Ü¹Þ°í 6°³¿ù ÀÌ»ó °øº¹ Ç÷´ç Á¶ÀýÀ» À§ÇØ Ä¡·á ¹Þ°í ÀÖ´Â È¯ÀÚ
¡¤ ´çÈ­ Ç÷»ö¼Ò HbA1c°¡ 6.5% ÀÌ»ó, 11.0% ÀÌÇÏÀÎ È¯ÀÚ

- º» ¿¬±¸ÀÇ Á¦¿Ü ±âÁØ
¡¤ ÃÖ±Ù 2°³¿ù µ¿¾È Ç÷´ç Á¶ÀýÀ» À§ÇØ ¾à¹° ¿ë·®À» Áõ·®Çß°Å³ª »õ·Î¿î ¾à¹°À» Ãß°¡ÇÑ È¯ÀÚ
¡¤ Èí¿¬À» ÇÏ°Å³ª, Áö¼ÓÀûÀÎ À½ÁÖ¸¦ ½ÃÇàÇÏ´Â °æ¿ì
¡¤ Æò¼Ò¿¡µµ Çö¹ÌÃ¤½ÄÀ» ½ÇÇàÇÏ°í ÀÖ´Â °æ¿ì
¡¤ ÇöÀç ÀÓ½Å ÁßÀÌ°Å³ª ÀÓ½Å °èÈ¹ÀÌ ÀÖ´Â °æ¿ì
¡¤ ¸¸¼º ½ÅºÎÀü µî ´ç´¢º´À¸·Î ÀÎÇÑ ½É°¢ÇÑ ÇÕº´ÁõÀÌ ÀÖ´Â °æ¿ì
  (¡Ø´Ü, ÇãÇ÷¼º ½ÉÁúÈ¯, ³úÁ¹ÁßÀº Á¦¿Ü ±âÁØ¿¡ ÇØ´çÇÏÁö ¾ÊÀ½)

- ¿¬±¸ Âü¿©ÀÚÀÇ Áßµµ Å»¶ô ±âÁØ
¡¤ ¿¬±¸ Âü¿©ÀÚ°¡ ½ÃÇèÀ» Áö¼ÓÇÒ °æ¿ì ÇØ°¡ µÈ´Ù°í ÁÖÄ¡ÀÇ»ç°¡ ÆÇ´ÜÇÏ´Â °æ¿ì


- ¿¬±¸ ´ë»óÀÚ ¼ö¿Í »êÁ¤ ±Ù°Å
  º» ¿¬±¸´Â Çö¹ÌÃ¤½Ä±º°ú ´ç´¢º´ ½ÄÀÌ±ºÀÇ µÎ Áý´Ü »çÀÌÀÇ Æò±Õº¯È­¸¦ ºñ±³ÇÏ´Â ¸ðµ¨·Î¼­, ÀÏÂ÷Æò°¡º¯¼ö´Â ÀûÇ÷±¸³» ´çÈ­Ç÷»ö¼Ò(HbA1c)ÀÇ º¯È­·®À¸·Î ÇÏ¿´´Ù. ¼±Çà ¿¬±¸(2-5)¸¦ ÂüÁ¶ÇÏ¿© °¢ ±×·ì°£ÀÇ ÀûÇ÷±¸³» ´çÈ­Ç÷»ö¼Ò(HbA1c) º¯È­·®ÀÇ Â÷ÀÌ 0.65, Ç¥ÁØÆíÂ÷ 1.0À» ÀÌ¿ëÇÏ¿© ¿¬±¸ ´ë»óÀÚ¼ö¸¦ »êÁ¤ÇÏ¿´´Ù. ¿¬±¸ ´ë»óÀÚ ¼ö »êÁ¤¿¡´Â 5%ÀÇ À¯ÀÇ¼öÁØ°ú 80%ÀÇ °ËÁ¤·ÂÀ» °¡Áö°í ¾çÃø°ËÁ¤À» ÇÏ´Â °ÍÀ¸·Î ÇÏ¿´´Ù. ºÐ¼® °á°ú °¢ ±º´ç 37¸íÀÇ ´ë»óÀÚ°¡ ÇÊ¿äÇÑ °ÍÀ¸·Î ³ªÅ¸³ª, ¼±Çà ¿¬±¸ÀÇ Áßµµ Å»¶ô·ü 30%¸¦ °í·ÁÇÏ¿© °¢ ±º¸¶´Ù 53¸í, ÃÑ 106¸íÀÇ ¿¬±¸ ´ë»óÀÚ ¼ö¸¦ »êÁ¤ÇÏ¿´´Ù.

  ¿¬±¸ ´ë»óÀÚ ¼ö¸¦ »êÁ¤ÇÏ´Â µ¥¿¡´Â ´ÙÀ½ÀÇ °ø½ÄÀÌ ÀÌ¿ëµÇ¾ú´Ù.


 : ´ëÁ¶±ºÀÇ baseline°ú intervention ÈÄ ÃøÁ¤º¯¼öÀÇ Æò±Õº¯È­
 : ½ÇÇè±ºÀÇ baseline°ú intervention ÈÄ ÃøÁ¤º¯¼öÀÇ Æò±Õº¯È­
 : ÃøÁ¤º¯¼öÀÇ ºÐ»ê

7. ÀÓ»ó½ÃÇè ¹æ¹ý

1) º» ¿¬±¸´Â ÃÑ 2°³ÀÇ ±ºÀ¸·Î ±¸¼ºµÈ´Ù.
¡¤ ±âÁ¸¿¡ »ç¿ëÇÏ´ø ¾à¹°¿ä¹ý + ´ëÇÑ ´ç´¢º´ÇÐÈ¸ ±ÇÀå ´ç´¢º´ ½ÄÀÌ
¡¤ ±âÁ¸¿¡ »ç¿ëÇÏ´ø ¾à¹°¿ä¹ý + Çö¹ÌÃ¤½Ä

2) ÀüÇâÀû, ¹«ÀÛÀ§Àû ÀÓ»ó½ÃÇè
¡¤ ¿¬±¸ ´ë»óÀÚµéÀ» ¸ðÁýÇÑ ´ÙÀ½ HbA1c 8.0%¸¦ cut off value·Î ÇÏ¿© 2°³ÀÇ ÃþÀ» ¸¸µç ÈÄ °¢°¢ÀÇ ±ºÀ¸·Î ¹«ÀÛÀ§·Î ÇÒ´çÀ» ÇÑ´Ù. ½ÄÀÌÀÇ °æ¿ì È¯ÀÚ°¡ Á÷Á¢ ¼öÇàÇØ¾ß ÇÏ¹Ç·Î ´«°¡¸²¹ýÀº »ç¿ëÇÒ ¼ö ¾ø´Ù.
¡¤ ¿¬±¸ ´ë»óÀÚÀÇ ÃþÈ­¸¦ À§ÇÑ HbA1c´Â µ¿ÀÇ¼­ ÀÛ¼º½Ã (1) ÃÖ±Ù 7ÀÏ ÀÌ³»ÀÇ HbA1c °á°ú°¡ ÀÖÀ» °æ¿ì ÀÌ¸¦ ÂüÁ¶ÇÔ. (2) ¿Ü·¡ Áø·á ÈÄ Áø·áÀÇ»çÀÇ ¿ø³»Ã³¹æÀÌ ÀÖÀ» °æ¿ì ÀÌ °á°ú¸¦ ÂüÁ¶ÇÔ. (3) (1) ¶Ç´Â (2)¸¦ ÀÌ¿ëÇÒ ¼ö ¾ø´Â °æ¿ì Ç÷¾×À» Ã¤ÃëÇÏ¿© ¿ÜºÎ±â°ü¿¡ ÀÇ·ÚÇÑ ÈÄ ÀÌ °á°ú¸¦ ÂüÁ¶ÇÔ. 
¡¤ °¢°¢ÀÇ ½ÄÀÌ¿ä¹ýÀº ÃÑ 12ÁÖ°£ ½ÃÇàÇÑ´Ù.
¡¤ ¿¬±¸ ½ÃÀÛ ÈÄ 4ÁÖ, 12ÁÖ¿¡ ÃßÀû°üÂûÀ» ½ÃÇàÇÑ´Ù.


3) ¹«ÀÛÀ§ ¹èÁ¤
¡¤ Stratified Block RandomizationÀ» ½ÃÇàÇÑ´Ù.
¡¤ HbA1c value 8.0%¸¦ cutoff value·Î ÇÏ¿© 8.0%º¸´Ù ³·Àº Ãþ(<8.0%), ³ôÀº Ãþ(¡Ã8.0%)À¸·Î ÃþÈ­ÇÑ´Ù.
¡¤ °¢ ÃþÈ­ÇÑ 2°³ÀÇ strata³»¿¡¼­ °¢°¢ Block RandomizationÀ» ½ÃÇàÇÑ´Ù.

4) °¢ ±ºÀÇ ½ÄÀÌÁ¶Àý ¹æ¹ý
¡¤ ÀÌ¹ø ¿¬±¸ÀÇ ¸ñÀûÀº ½ÄÀÌ ¿ä¹ýÀÇ È¿°ú¸¦ ¾Ë¾Æº¸´Â °ÍÀÌ¹Ç·Î, ¸ðµç ¿¬±¸ Âü¿©ÀÚµéÀº ¿îµ¿ ½À°üÀÇ º¯È­¸¦ ±Ç°í ¹ÞÁö ¾Ê°í, Æò¼ÒÀÇ ¿îµ¿ ½À°üÀ» ±×´ë·Î À¯ÁöÇÏµµ·Ï ÇÑ´Ù. ½ÄÀÌ¿ä¹ýÀº ÃÑ 12ÁÖ µ¿¾È ÁøÇàÇÑ´Ù.

-½ÃÇè±º(Çö¹Ì Ã¤½Ä)
	¸ðµç °î·ù´Â µµÁ¤À» ÇÏÁö ¾ÊÀº whole grainÀ» ¼·ÃëÇÏµµ·Ï ÇÔ
	Èò½Ò, º¸¸®½ÒÀº ¼·Ãë°¡ ±ÝÁöµÇ¸ç, 100% Çö¹Ì·Î ´ëÃ¼
	½Ò°¡·ç, ¹Ð°¡·ç µîÀ» ÅëÇØ °¡°øµÈ ½ÄÇ° ¶ÇÇÑ ¼·Ãë°¡ ±ÝÁöµÊ
	¸ðµç µ¿¹°¼º ½ÄÇ°(À°·ù, °¡±Ý·ù, »ý¼±, À¯Á¦Ç° ¹× ´Þ°¿)Àº ¼·Ãë°¡ ±ÝÁöµÊ
	³·Àº glycemic indexÀÇ ½ÄÇ°(¿¹, Äá·ù³ª ÄáÀ¸·Î ¸¸µç ½ÄÇ°, ³ì»öÃ¤¼Ò)ÀÇ ¼·Ãë¸¦ ±ÇÀå
	¿¬±¸ ´ë»óÀÚµéÀº ¸Ô¾î¾ß µÉ À½½ÄÀ» ±³À°¹Þ±âº¸´Ù´Â, ¸ÔÁö ¸»¾Æ¾ß ÇÒ À½½Ä À§ÁÖ·Î ±³À° ¹Þ°Ô µÊ
	½Ä»çÀÇ ¾ç, È½¼ö, ½Ã°£Àº Á¦ÇÑÀ» µÎÁö ¾ÊÀ½(¿­·® Á¦ÇÑÀ» µÎÁö ¾ÊÀ½)

-´ëÁ¶±º(´ç´¢º´ ½ÄÀÌ)
	´ëÇÑ´ç´¢º´ÇÐÈ¸ÀÇ Áø·áÁöÄ§ 2011 ¹× ´ëÇÑ¿µ¾ç»çÇùÈ¸ÀÇ ÀÓ»ó¿µ¾ç°ü¸®ÁöÄ§¼­(3ÆÇ)ÀÇ °¡ÀÌµå¶óÀÎÀ» µû¸§
	Ã¼Áß °¨·®ÀÌ ÀÎ½¶¸° ÀúÇ×¼ºÀ» °³¼±½ÃÅ°¹Ç·Î Ã¼Áß °¨·®ÀÌ ±ÇÀåµÊ. ½Ä»çÁ¶ÀýÀ» ÅëÇØ Ä®·Î¸® ¼·Ãë¸¦ ÁÙÀÌ´Âµ¥ Ä®·Î¸® ¼·Ãë·®Àº °³°³ÀÎÀÇ Ã¼°Ý, ½ÅÃ¼È°µ¿ Á¤µµ, Ã¼ÁßÁ¶ÀýÀÇ ÇÊ¿ä¼º, Æò¼ÒÀÇ Ä®·Î¸® ¼·Ãë·®, °³ÀÎÀÇ ¼øÀÀµµ µîÀ» °í·ÁÇÏ¿© ÀûÁ¤¼öÁØÀ¸·Î °áÁ¤ÇÏ¿©¾ß ÇÔ
	ÀÏ¹ÝÀûÀ¸·Î ¼ºÀÎ ´ç´¢º´È¯ÀÚÀÇ °æ¿ì ÀÏÀÏ Ä®·Î¸® ¼·Ãë·®À» Á¦ÇÑÇÏ´Âµ¥, º¸Åë Ä®·Î¸® ÇÊ¿ä·® °è»ê½Ã ´ëÇÑ´ç´¢º´ÇÐÈ¸ ±³À°À§¿øÈ¸ÀÇ ´ç´¢º´ ±³À°ÁöÄ§¼­(2006)¸¦ µû¸§
	Åº¼öÈ­¹°Àº ÃÑ ¿¡³ÊÁöÀÇ 50-60%¸¦ ¼·ÃëÇÏ¸ç Àü°î·ù, °úÀÏ, Ã¤¼Ò, ÀúÁö¹æ¿ìÀ¯°¡ Æ÷ÇÔµÈ °Ç°­ÇÑ ½Ä»ç·Î ±¸¼ºµÇ¾î¾ß ÇÔ
	´ç´¢º´È¯ÀÚ¿¡¼­ ½Å±â´ÉÀÌ Á¤»óÀÌ¸é º¸ÅëÀÇ ´Ü¹éÁú ¼·Ãë·®(ÃÑ ¿¡³ÊÁöÀÇ 15-20%)À» À¯ÁöÇÔ
	Áö¹æÀÇ ¼·Ãë·®Àº ÃÑ ¿¡³ÊÁöÀÇ 25% ÀÌ³»·Î ÇÏ¸ç, Æ÷È­Áö¹æÀÇ ¼·Ãë´Â ÃÑ ¿¡³ÊÁöÀÇ 7% ¹Ì¸¸, Æ®·£½ºÁö¹æ ¼·Ãë´Â ÃÖ¼ÒÈ­ÇÏ°í ÄÝ·¹½ºÅ×·Ñ ¼·Ãë´Â 1ÀÏ 200mg ÀÌÇÏ·Î ÇÔ

-½ÄÀÌ±³À° °èÈ¹ ¹× ±âÅ¸»çÇ×
	ÀÓ»ó½ÃÇè Âü¿©ÀÚµé¿¡°Ô º°µµÀÇ ½Ä»ç°¡ Á¦°øµÇÁö´Â ¾ÊÀ¸¸ç, Âü¿©ÀÚµéÀÌ ÀÚÀ¯·Ó°Ô »ýÈ°ÇÏµµ·Ï ÇÔ
	ÀÓ»ó½ÃÇè ±â°£µ¿¾È º¸Á¶ºñÅ¸¹Î ¼·Ãë´Â º°µµ·Î ÇÏÁö ¾ÊÀ½
	ÀÓ»ó½ÃÇè Âü¿© ±â°£µ¿¾È µÎ ±º ¸ðµÎ ¿îµ¿½À°üÀº Æò¼Ò¿Í ´Ù¸§¾øÀÌ À¯ÁöÇÏ¸ç º¯µ¿ÀÌ ¾øµµ·Ï ÇÔ
	°¢ ±ºÀ¸·Î ÇÒ´çµÈ È¯ÀÚµéÀº ¿¬±¸½ÃÀÛ½ÃÁ¡¿¡ ÀÚ½ÅÀÌ ¹èÁ¤¹ÞÀº ½ÄÀÌÇÁ·Î±×·¥¿¡ ´ëÇØ º» ¿¬±¸¿¡ µî·ÏµÈ ¿µ¾ç»ç·ÎºÎÅÍ 1½Ã°£ µ¿¾È ½ÄÀÌ±³À°À» ¹Þ°í ½º½º·Î ÀûÀýÇÑ ½ÄÀÌ°èÈ¹À» ¼¼¿ï ¼ö ÀÖµµ·Ï ÇÔ
	ÀÌÈÄ¿¡´Â ÁÖ 1È¸ ¿µ¾ç»ç°¡ ¹Ì¸® ¿¹°íÇÏÁö ¾Ê°í ÀüÈ­¸¦ °É¾î 24½Ã°£ È¸»ó¹ýÀ» ÀÌ¿ëÇÑ ½ÄÀÌÁ¶»ç¸¦ ½ÃÇàÇÏ°í ½ÄÀÌÁ¶Àý ¹× Á¶¸®ÁöÄ§¿¡ ´ëÇÑ »ó´ãÀ» Á¦°ø
	µÎ ±º¿¡¼­ »ó´ã ½Ã°£°ú »ó´ã Ç×¸ñÀº °°À¸¸ç, ¼¼ºÎÀûÀÎ ½ÄÀÌ¿ä¹ý ÁöÄ§¸¸ Â÷ÀÌ°¡ ÀÖÀ½. ¶ÇÇÑ °¢ ±ºÀÇ ´ã´çÀÚ´Â ¾î´À ½ÄÀÌ¸¦ ¼±È£ÇÑ´Ù´Â ³»¿ëÀ» ¾ð±ÞÇÏÁö ¸øÇÔ 

5) °üÂûÇ×¸ñ, °üÂû°Ë»ç¹æ¹ý ¹× ÀÓ»ó°Ë»çÇ×¸ñ
l	½ÅÃ¼°èÃø ¹× »ýÃ¼ÁöÇ¥ ÃøÁ¤: ½ÅÀå, Ã¼Áß, Çã¸®µÑ·¹, ¾ûµ¢ÀÌ µÑ·¹, Ç÷¾Ð ÃøÁ¤
l	Ç÷¾×°Ë»ç: °øº¹Ç÷´ç(Fasting plasma glucose), Áß¼ºÁö¹æ(Triglyceride), ÄÝ·¹½ºÅ×·Ñ(Total cholesterol, HDL-C, LDL-C), GGT, C-reactive protein, Insulin, AST, ALT, ÀûÇ÷±¸³» ´çÈ­Ç÷»ö¼Ò(HbA1c), Ç÷Ã» POPs
l	¼Òº¯°Ë»ç: Urine Glucose, Protein, RBC, WBC  
l	ºÐº¯°Ë»ç: ´ëº¯³» POPs, Àå³» ¼¼±ÕÃÑ
l	¼³¹®Á¶»ç: ½ÄÀÌ ¹× ±âÅ¸ °Ç°­ÇàÅÂ Á¶»ç
l	½ÄÀÌ¼øÀÀµµ Æò°¡

6) ´ë»óÀÚÀÇ ÃßÀû°üÂû

(1) ½ÄÀÌÁ¶ÀýÀÇ Á¡°Ë
¡¤ 1ÁÖÀÏ¿¡ 1¹ø¾¿ ¿µ¾ç»ç°¡ ÀüÈ­¸¦ ÅëÇØ ½ÄÀÌ Á¶Àý °úÁ¤¿¡ ´ëÇÑ Á¡°ËÀ» ÇÑ´Ù.
¡¤ 1´Þ¿¡ ÃÑ 4È¸(ÁÖÁß 3È¸, ÁÖ¸» 1È¸)ÀÇ 1ÀÏ µ¿¾È ¸ÔÀº À½½Ä¿¡ ´ëÇØ 24½Ã°£ È¸»ó¹ýÀ» »ç¿ëÇÑ´Ù.
¡¤ ¸ÅÀÏ¸ÅÀÏ ½ÄÀÌÁ¶Àý ½ÇÃµÁ¤µµ¿¡ ´ëÇØ ¿¬±¸ Âü¿©ÀÚ ½º½º·Î ÀÚ°¡ Á¡°ËÀ» ÇÏµµ·Ï ÇÑ´Ù.
¡¤ ÀüÈ­ ÅëÈ­½Ã È¯ÀÚ°¡ ½ÄÀÌ¿ä¹ý ÁøÇàÁß »ý±â´Â ÀÇ¹®Á¡À» »ó´ãÇØÁÖµµ·Ï ÇÑ´Ù.

(2) ½ÅÃ¼°èÃø, ¹× ÀÓ»ó°Ë»çÀÇ ÃßÀû°üÂû
¡¤ 4ÁÖ, 12ÁÖ¿¡ ½ÅÃ¼°èÃø°ú ÀÓ»ó°Ë»ç¿¡ ´ëÇÑ ÃßÀû°üÂû ¹× ¿µ¾ç»ç¿ÍÀÇ ¸é´ãÀ» ÇÔ²² ½Ç½ÃÇÑ´Ù.
¡¤ ´ã´ç ÀÇ»çÀÇ ¸é´ãÀº 12ÁÖ¿¡ ½Ç½ÃÇÏ¸ç, ÇÊ¿äÇÑ °æ¿ì 4ÁÖ¿¡ ¼±ÅÃÀûÀ¸·Î ÀÇ»ç ¸é´ãÀ» ½Ç½ÃÇÑ´Ù. 

(3) ¼øÀÀµµ Æò°¡
¡¤ ÀÚ°¡ Æò°¡°¡ 5Á¡ ¹Ì¸¸ÀÎ °æ¿ì ½ÄÀÌ¿ä¹ýÀ» Á¦´ë·Î ¼öÇàÇÏÁö ¾ÊÀº °ÍÀ¸·Î ÆÇ´ÜÇÑ´Ù.
¡¤ ½ÄÀÌ¿ä¹ýÀ» ÃÑ 12ÁÖ Áß 6ÁÖ ÀÌ»óÀ» Á¦´ë·Î ¼öÇàÇÏÁö ¸øÇßÀ» °æ¿ì ¼øÀÀµµ°¡ ¶³¾îÁö´Â °ÍÀ¸·Î ÇÑ´Ù.
 
(4) ´ç´¢Ä¡·áÀÇ Áö¼Ó
¡¤ ¿¬±¸ Âü¿©ÀÚµéÀº ¿¬±¸ ±â°£ÀÎ 12ÁÖ µ¿¾È ¾à ¿ë·®ÀÌ³ª Á¾·ùÀÇ Á¶Àý¾øÀÌ Æò¼Ò ¹Þ´ø ´ç´¢ Ä¡·á¸¦ Áö¼ÓÇÏµÇ, ÀÇ»çÀÇ Áø·á °á°ú ¾à ¿ë·®À» °¨¼Ò½ÃÄÑ¾ß ÇÏ´Â °æ¿ì ÀÌ¸¦ °¡´ÉÇÏµµ·Ï ÇÔ. 

8. È¿°úÆÇÁ¤±âÁØ, Æò°¡¹æ¹ý ¹× ÇØ¼®¹æ¹ý

- À¯È¿¼º Æò°¡
¡¤ Primary Endpoint : ¿¬±¸½ÃÀÛ½ÃÁ¡°ú Á¾·á½ÃÁ¡¿¡¼­ÀÇ HbA1c º¯È­·®
¡¤ Secondary Endpoint : Ã¼Áß, BMI, FBS, TG, TC, GGT, Insulin, blood pressure

- Åë°èºÐ¼®¹æ¹ý
¡¤ Çö¹ÌÃ¤½Ä±º°ú ´ç´¢º´ ½ÄÀÌ±º°£ÀÇ HbA1cÀÇ º¯È­·®À» Æò°¡(Primary endpoint – RM-ANOVA »ç¿ë)

9. ¾ÈÁ¤¼º Æò°¡±âÁØ, Æò°¡¹æ¹ý ¹× º¸°í¹æ¹ý

- ¾ÈÁ¤¼º Æò°¡
¡¤ ½ÄÀÌ¿ä¹ýÀÇ Æ¯º°ÇÑ ºÎÀÛ¿ëÀº °ÅÀÇ ¾ø´Ù.

10. ±âÅ¸ÀÓ»ó½ÃÇèÀ» ¾ÈÀüÇÏ°í °úÇÐÀûÀ¸·Î ½Ç½ÃÇÏ±â À§ÇÏ¿© ÇÊ¿äÇÑ »çÇ×

- ÇÇÇèÀÚ µî·Ï
¡¤ ¸ðµç ½ÃÇèÀÚ´Â °¢ ½ÃÇè±â°üÀÇ ÀÓ»ó½ÃÇè½É»çÀ§¿øÈ¸·ÎºÎÅÍ º» ½ÃÇè°èÈ¹¼­, µ¿ÀÇ¼­ µî¿¡ °üÇÑ ½ÂÀÎÀ» ¾òÀº ÈÄ¿¡ ÇÇÇèÀÚ¸¦ º» ÀÓ»ó½ÃÇè¿¡ ¸ðÁýÇÒ ¼ö ÀÖ´Ù. ¸ðµç ÇÇÇèÀÚ(¶Ç´Â ¹ýÀû ´ë¸®ÀÎ)¿¡°Ô´Â ÀÓ»ó½ÃÇè¿¡ Âü¿©ÇÏ±â ÀÌÀü¿¡ ¼­¸é µ¿ÀÇ¼­¿¡ ÀÚÇÊ·Î ¼­¸íÇÏ°í ³¯Â¥¸¦ ±â·ÏÇÏµµ·Ï ÇÏ¿© ÀÚ¹ßÀûÀ¸·Î ÀÓ»ó½ÃÇè¿¡ Âü¿©ÇÔÀ» È®ÀÎÇØ¾ß ÇÑ´Ù.

- ÇÇÇèÀÚ µ¿ÀÇ
¡¤ ÇÇÇèÀÚ¸¦ ÀÓ»ó½ÃÇè¿¡ Âü¿©½ÃÅ°±â Àü, ½ÃÇè´ã´çÀÚ´Â ÇØ´ç ÇÇÇèÀÚ ¹× ÇÊ¿äÇÑ °æ¿ì ¹ýÀû º¸È£ÀÚ¿¡°Ô º» ÀÓ»ó½ÃÇè¿¡ ´ëÇØ ¸ñÀû, ¹æ¹ý ¾òÀ» ¼ö ÀÖ´Â ÇýÅÃ ¹× À§Çè, ÇÇÇèÀÚÀÇ ±Ç¸®, ´ëÃ¼¿ä¹ý µî¿¡ ´ëÇØ ÀÚ¼¼È÷ ¼³¸íÇÏ°í ¹Ýµå½Ã ¹®¼­µ¿ÀÇ¸¦ ¾ò¾î¾ß ÇÑ´Ù. µ¿ÀÇ¼­´Â 2ºÎ¸¦ ÀÛ¼ºÇÏ¿© ÇÑ ºÎ´Â ÇÇÇèÀÚ ¶Ç´Â ¹ýÀû º¸È£ÀÚ, ÇÑ ºÎ´Â ½ÃÇè´ã´çÀÚ°¡ º¸°üÇÑ´Ù. ½ÃÇè´ã´çÀÚ´Â ÇÇÇèÀÚÀÇ µ¿ÀÇ¼­¸¦ ¹Ýµå½Ã Áõ·Ê±â·Ï¼­¿Í ÇÔ²² º¸°üÇØ¾ß ÇÏ°í µ¿ÀÇ¼­¸¦ ¹ÞÀº ³¯Â¥¸¦ Áõ·Ê±â·Ï¼­¿¡ ±â·ÏÇÑ´Ù.

- ÀÓ»ó½ÃÇè ½É»çÀ§¿øÈ¸
¡¤ ½ÃÇèÃ¥ÀÓÀÚ´Â ½É»çÀ§¿øÈ¸(ÀÌÇÏ IRB)¿¡ ÀÓ»ó½ÃÇè °èÈ¹¼­, ÇÇÇèÀÚ µ¿ÀÇ ¾ç½Ä µîÀ» Á¦ÃâÇÏ¿© ¼­¸é ½ÂÀÎÀ» ¹Þµµ·Ï ÇÑ´Ù. ½ÃÇèÀÚ´Â ½ÃÇè°èÈ¹¼­ÀÇ º¯°æÀÌ³ª ÇÇÇèÀÚ µ¿ÀÇ¼­ ¼­·ù¿¡ ¼öÁ¤ÀÌ ÀÖ´Â °æ¿ì IRB¿¡ ¾Ë¸®°í ÇÊ¿äÇÑ °æ¿ì ½ÂÀÎÀ» ¹Þ¾Æ¾ß ÇÑ´Ù. ½ÃÇèÀÚ´Â ÀÓ»ó½ÃÇè ±â°ü¿¡¼­ Áß´ëÇÑ ÀÌ»ó¹ÝÀÀÀÌ ¹ß»ýÇÏ¿´°Å³ª ½ÃÇè°èÈ¹¼­´ë·Î ½ÃÇèÀÌ ÁøÇàµÇÁö ¾Ê¾ÒÀ» °æ¿ì, ±×¸®°í ÀÇ·ÚÀÚ·ÎºÎÅÍ ±âÅ¸ ÀÌ»ó¹ÝÀÀÀÌ º¸°íµÇ¾úÀ» °æ¿ì IRB¿¡ ¾Ë·Á¾ß ÇÑ´Ù. IRB´Â ÀÓ»ó½ÃÇèÀÌ À±¸®ÀûÀÎ Ãø¸é¿¡¼­ ÀçÆò°¡°¡ ÇÊ¿äÇÑÁö¿¡ ´ëÇÑ °ßÁ¦¸¦ ÇÇ·ÂÇÒ ¼ö ÀÖ´Ù. 


Âü°í ¹®Çå

1. American Diabetes Association. Nutrition Recommendations and
Interventions for Diabetes. A position statement of the American Diabetes Association. Dibetes Care, Volume 31, Supplement 1, January 2008
2. Barnard ND, Scialli AR, Turner-McGrievy G, Lanou AJ, Glass J. The effects of a low-fat, plant-based dietary intervention on body weight, metabolism, and insulin sensitivity. Am J Med. 2005;118:991–.997
3. Turner-McGrievy GM, Barnard ND, Scialli AR. A two-year randomized weight loss trial comparing a vegan diet to a more moderate low-fat diet. Obesity (Silver Spring). 2007;15:2276–. 2281.
4. Barnard ND, Cohen J, Jenkins DJ, et al. A low-fat vegan diet improves glycemic control and cardiovascular risk factors in a randomized clinical trial in individuals with type 2 diabetes. Diabetes Care. 2006;29:1777–.1783.
5. Neal D Barnard, Joshua Cohen, David JA Jenkins, Gabrielle Turner-McGrievy, Lise Gloede, Amber Green, and Hope Ferdowsian.A low-fat vegan diet and a conventional diabetes diet in the treatment of type 2 diabetes: a randomized, controlled, 74-wk clinical trial. Am J Clin Nutr 2009;89(suppl):1588S–-96S 

[¼­½Ä 11] 

ÇÇÇèÀÚ ¼³¸í¹®

ÀÓ»ó½ÃÇè Á¦¸ñ : Á¦ 2Çü ´ç´¢º´¿¡¼­ Ã¤½ÄÀÇ ÀÓ»óÈ¿°ú Æò°¡ ¹× ÀÜ·ù¼º À¯±â¿À¿°¹°Áú(Persistent Organic Pollutants, POPs) °ü·Ã ±âÀü ¿¬±¸

ÀÓ»ó½ÃÇè °èÈ¹¼­ ¹øÈ£ : 
½ÃÇè Ã¥ÀÓÀÚ ¼º¸í : ÀÌ´öÈñ
ÁÖ¼Ò : ´ë±¸±¤¿ª½Ã Áß±¸ µ¿ÀÎ2°¡ °æºÏ´ëÇÐ±³ ÀÇÇÐÀü¹®´ëÇÐ¿ø ¿¹¹æÀÇÇÐ±³½Ç ¿ì):700-421 
ÀüÈ­¹øÈ£ : 053)420-

ÇÇÇèÀÚ ¼º¸í : 
»ý³â¿ùÀÏ : ³â ¿ù ÀÏ
ÁÖ¼Ò :

º» ¿¬±¸´Â °æºÏ´ëÇÐ±³ ÀÇÇÐÀü¹®´ëÇÐ¿ø ¿¹¹æÀÇÇÐ±³½Ç, °æºÏ´ëÇÐ±³ º´¿ø ³»ºÐºñ³»°ú ÇÔ²² ¼öÇàÇÏ´Â ¿¬±¸ »ç¾÷À¸·Î½á ´ç´¢ È¯ÀÚ¿¡¼­ÀÇ ½ÄÀÌ¿ä¹ýÀÌ Ç÷´ç Á¶Àý, Ã¼Áß ¹× °Ç°­ÁöÇ¥¿¡ ¹ÌÄ¡´Â ¿µÇâÀ» ¾Ë¾Æº¸±â À§ÇÑ °ÍÀÔ´Ï´Ù.
Á¦ 2Çü ´ç´¢º´ÀÇ °ü¸®, ÇÕº´ÁõÀÇ ¿¹¹æ ¹× Áö¿¬¿¡ ÀÖ¾î¼­ ½ÄÀÌ¿ä¹ýÀÇ Áß¿ä¼ºÀº ¸Å¿ì Àß ¾Ë·ÁÁ® ÀÖ½À´Ï´Ù. ´ëÇÑ ´ç´¢º´ ÇÐÈ¸¿¡¼­´Â ´ç´¢º´ °ü¸®¸¦ À§ÇÑ ½ÄÀÌ¿ä¹ýÀ¸·Î Åº¼öÈ­¹° ¼·Ãë ¹× Æ÷È­ Áö¹æ°ú Æ®·£½º Áö¹æ, ÄÝ·¹½ºÅ×·ÑÀÇ ¼·Ãë Á¦ÇÑ, ±×¸®°í °úÃ¼Áß È¯ÀÚ¿¡¼­ÀÇ ¿­·® ¼·Ãë Á¦ÇÑÀ» ±Ç°íÇÏ°í ÀÖÀ¸¸ç, ÇöÀç ´ëºÎºÐÀÇ º´¿ø¿¡¼­ ÀÌ¿Í ÇÔ²² °³ÀÎÀÇ ÀÇÇÐÀû »óÅÂ¿Í »ýÈ°¹æ½Ä, À½½Ä ±âÈ£µµ¿¡ ¸ÂÃá ½ÄÀÌ ¿ä¹ýÀ» ´ç´¢º´ È¯ÀÚ¿¡°Ô ±Ç°íÇÏ°í ÀÖ½À´Ï´Ù. ÇÏÁö¸¸, ÃÖ±Ù ±¹¿Ü¿¡¼­ ½ÃÇàµÈ ¸î¸î ¹«ÀÛÀ§ÀÓ»ó½ÃÇè¿¬±¸¿¡¼­´Â ¿­·®À» Á¦ÇÑÇÏÁö ¾Ê°í µ¿¹°¼º ½ÄÇ°À» ¸ÔÁö ¾Êµµ·Ï ÇÏ´Â Ã¤½ÄÀ» ½ÃÇàÇÑ ±ºÀÌ ±âÁ¸ÀÇ ¹Ì±¹ ´ç´¢º´ÇÐÈ¸¿¡¼­ ±ÇÀåÇÏ´Â ´ç´¢º´ ½ÄÀÌ ¿ä¹ý±º¿¡ ºñÇØ Ç÷´ç°ü¸®¿¡ ÀÖ¾î ´õ ¿ì¼öÇÏ´Ù´Â ¿¬±¸°á°úµéÀ» º¸°íÇÏ°í ÀÖ½À´Ï´Ù. 

±×·¯³ª, ÇöÀç·Î¼­´Â ÀÌ·¯ÇÑ ÃÖ±ÙÀÇ ¸î¸î ¿¬±¸ °á°úµé¸¸À¸·Î Á¤È®È÷ ´ç´¢º´ÇÐÈ¸¿¡¼­ ±ÇÀåÇÏ´Â ´ç´¢º´ ½ÄÀÌ¿ä¹ý°ú Ã¤½Ä Áß ¾î´À ½ÄÀÌ ¿ä¹ýÀÌ ´ç´¢º´ °ü¸® ¹× ÇÕº´Áõ ¹ß»ý ¿¹¹æ¿¡ ´õ È¿°úÀûÀÎÁö ¾Ë ¼ö ¾ø½À´Ï´Ù. µû¶ó¼­, º» ¿¬±¸¿¡¼­´Â ¿¬±¸ Âü°¡ÀÚµéÀ» ¸ðÁýÇÏ¿© ´ëÇÑ ´ç´¢º´ ÇÐÈ¸ ±ÇÀå ´ç´¢º´ ½ÄÀÌ±º°ú Çö¹ÌÃ¤½Ä±ºÀ¸·Î ±¸ºÐÇÏ¿© °¢ ½ÄÀÌ¿ä¹ýÀÇ È¿°ú¸¦ ºñ±³ÇØ º¸°íÀÚ ÇÕ´Ï´Ù. 
º» ¿¬±¸¿¡ Âü¿© ÈÄ ½ÇÃµÇØ¾ß ÇÒ ½ÄÀÌ¿ä¹ýÀº ¹«ÀÛÀ§·Î ³ª´©¾îÁö°Ô µÇ°í ´©°¡ ¾î¶² ½ÄÀÌ¿ä¹ýÀ» ½ÃÇàÇÏ´À³Ä ÇÏ´Â °ÍÀº ¿ì¿¬È÷ °áÁ¤µÇ´Â °ÍÀÌÁö ´©±¸¿¡°Ô Æ¯º°ÇÑ Á¡ÀÌ ÀÖ°Å³ª ¹®Á¦°¡ ÀÖ¾î¼­ ÀÇµµÀûÀ¸·Î ¾î¶² ½ÄÀÌ¿ä¹ýÀ» °áÁ¤ÇÏ´Â °ÍÀº ¾Æ´Õ´Ï´Ù. ÀÌ ¿¬±¸´Â 12ÁÖ µ¿¾È ÁøÇàµÇ´Âµ¥, ÀÌ µ¿¾È ±ÍÇÏ²²¼­´Â ¹èÁ¤¹ÞÀº ½ÄÀÌ¿ä¹ýÀ» ½ÇÃµÇÏ½Ã°Ô µË´Ï´Ù. ±ÍÇÏ²²¼­´Â ¾ÕÀ¸·Î 0ÁÖ, 4ÁÖ, 12ÁÖ¿¡ ÇÑ¹ø¾¿ º´¿ø¿¡ ¿À¼Å¼­ ¿µ¾ç»ç ¹× ´ã´çÀÇ»ç¿Í ¸é´ãÀ» °¡Áö°Ô µÇ¸ç, ½ÅÃ¼°Ë»ç, Ç÷¾×°Ë»ç, ºÐº¯°Ë»ç, ¼Òº¯°Ë»ç¸¦ ÇÏ°Ô µË´Ï´Ù. ÀÌ¶§ °Ë»ç¸¦ À§ÇØ¼­ »Ì´Â Ç÷¾×·®Àº ¾à 10-25mL Á¤µµ·Î Ç÷´ç, ÁöÁú°Ë»ç, °£±â´É°Ë»ç µîÀ» ¼öÇàÇÏ°Ô µË´Ï´Ù. ±ÍÇÏ²²¼­´Â ÀÌ·¯ÇÑ °Ë»ç¸¦ À§ÇÑ Ãß°¡ÀÇ ºñ¿ëÀ» ÁöºÒÇÏ½Ç ÇÊ¿ä°¡ ¾ø½À´Ï´Ù.
½ÄÀÌ ¿ä¹ýÀ¸·Î ÀÎÇÑ ºÎÀÛ¿ëÀº °ÅÀÇ ¾ø´Â ÆíÀ¸·Î ÀÌ¿¡ ´ëÇØ¼­´Â °ÆÁ¤À» ÇÏÁö ¾ÊÀ¸¼Åµµ µÇ¸ç, Ç÷¾× Ã¤Ãë½Ã ¾à°£ÀÇ ÅëÁõ ¹× ¾îÁö·¯¿ò µî °æ¹ÌÇÑ ºÎÀÛ¿ëÀÌ ³ªÅ¸³¯ ¼ö ÀÖ½À´Ï´Ù. ÀÌ´Â º¸Åë ÀÏ½ÃÀûÀÎ Áõ»óÀ¸·Î ±Ý¹æ ÇØ¼ÒµÉ °ÍÀÌÁö¸¸ ºÎÀÛ¿ëÀÇ Áõ»óÀÌ Áö¼ÓµÉ °æ¿ì °ü·Ã Áø·á ÈÄ ÇÊ¿äÇÑ Ä¡·á¸¦ ¹Þ°Ô ÇØµå¸± °ÍÀÔ´Ï´Ù.
ÀÌ ¿¬±¸¸¦ ÅëÇØ¼­ ±ÍÇÏ²²¼­´Â ¿¬±¸ ±â°£ µ¿¾È ´ã´ç¿µ¾ç»ç¸¦ ÅëÇÑ °³º° ¸ÂÃã ¿µ¾ç »ó´ãÀ» ¹ÞÀ¸½Ç ¼ö ÀÖÀ¸¸ç, ±ÍÇÏ²²¼­ ¿¬±¸¿¡ Âü¿©ÇÏ½Ã´Â 12ÁÖ µ¿¾ÈÀÇ °Ë»çºñ´Â ¹«·áÀÌ°í ¹æ¹®½Ã¸¶´Ù 5¸¸¿ø¾¿ ÃÑ 15¸¸¿øÀÇ ±³Åëºñ¸¦ Áö±Þ¹Þ°Ô µÉ °ÍÀÔ´Ï´Ù.
¿¬±¸¸¦ ÀÇ·ÚÇÑ ´ã´çÀÚ¿Í º´¿øÀÇ ¿¬±¸°£È£»ç, ¿¬±¸¿øÀÌ ¿¬±¸ ±â·Ï(ÀüÀÚÀÇ¹«±â·Ï Æ÷ÇÔ)À» ¿­¶÷ÇÒ ¼ö ÀÖ½À´Ï´Ù. ±×·¯³ª ¿¬±¸ ±â·ÏÀÇ ¿­¶÷Àº º» ¿¬±¸°úÁ¦¿¡ ±¹ÇÑµÇ¸ç ¿¬±¸ ¸ñÀûÀ¸·Î¸¸ »ç¿ëµÉ °ÍÀÔ´Ï´Ù. ¿¬±¸ ±â·ÏÀº ºñ¹Ð ¹øÈ£·Î °ü¸®µÇ¸ç ¿¬±¸ °á°ú¿¡ ´ëÇÑ ¾î¶² ÇÐ¼ú º¸°í¼­¿¡µµ ±ÍÇÏÀÇ ÀÌ¸§Àº ¹àÈ÷Áö ¾ÊÀ» °ÍÀÔ´Ï´Ù.
±ÍÇÏ²²¼­ ¿¬±¸¿¡ Âü¿©¸¦ °ÅÀýÇÏ¼Åµµ ±¦Âú°í ¿¬±¸¿¡ Âü¿©ÇÏ½Å ÈÄ¶óµµ ¾ðÁ¦µçÁö Ãë¼Ò¸¦ ÇÏ½Ç ¼ö°¡ ÀÖ½À´Ï´Ù. ¸¸ÀÏ ±ÍÇÏ²²¼­ °ÅÀýÇÏ°Å³ª Ãë¼ÒÇÏ½Ã´õ¶óµµ ÀúÈñ ÀÇ»ç³ª º´¿ø°úÀÇ °ü°è¿¡¼­ Á¶±ÝÀÇ ºÒÀÌÀÍµµ ¹ÞÁö ¾ÊÀ» °ÍÀÌ°í, Ç¥ÁØÀûÀÎ ´ç´¢ Ä¡·á¸¦ ÇØ µå¸± °ÍÀÔ´Ï´Ù. ¶Ç ±ÍÇÏ²²¼­ ¿øÇÏ½Å´Ù¸é, ÀÌ·± Ä¡·á¸¦ ÇÒ ¼ö ÀÖ´Â ´Ù¸¥ º´¿øÀÌ³ª ÀÇ»ç¸¦ ¼Ò°³ÇØ µå¸± ¼öµµ ÀÖ½À´Ï´Ù.


ÇÇÇèÀÚ ¼­¸é µ¿ÀÇ¼­

● º»ÀÎÀº ¡°Á¦ 2Çü ´ç´¢º´¿¡¼­ Ã¤½ÄÀÇ ÀÓ»óÈ¿°ú Æò°¡ ¹× ÀÜ·ù¼º À¯±â¿À¿°¹°Áú(Persistent Organic Pollutants, POPs) °ü·Ã ±âÀü ¿¬±¸¡±¿¡ ´ëÇÑ »ó±â ÇÇÇèÀÚ ¼³¸í¹®À» ÀÐ¾úÀ½À» ¼±¾ðÇÕ´Ï´Ù. 
● º»ÀÎÀº ÀÌ ¿¬±¸ÀÇ ¸ñÀû, °èÈ¹, °úÁ¤ ¹× À§Çè¿¡ °üÇÑ Á¤º¸¸¦ ÃæºÐÈ÷ Á¦°ø ¹Þ¾Ò½À´Ï´Ù. ÀÌ ¿¬±¸¿¡ ´ëÇÏ¿© º»ÀÎÀÇ Âü¿© Áß ±×¸®°í Âü¿© ÈÄ ¾ðÁ¦¶óµµ ¿¬±¸¿Í °ü·ÃµÈ ¼Õ»óÀÇ °æ¿ì»Ó¸¸ ¾Æ´Ï¶ó ¿¬±¸¿¡ ´ëÇÑ ¾î¶°ÇÑ Ãß°¡ÀûÀÎ Á¤º¸¶óµµ Á¦°øÇÒ Ã¥ÀÓÀÌ ¿¬±¸ÀÚ¿¡°Ô ÀÖ´Ù´Â °ÍÀ» º»ÀÎÀº ¾Ë°í ÀÖ½À´Ï´Ù. 
● º»ÀÎÀº ÀÌ ÀÓ»ó¿¬±¸·ÎºÎÅÍ ¾ò¾îÁø ¿¬±¸ ÀÚ·á°¡ ÀÌ ¿¬±¸ÀÇ °ü·Ã ¿ä¿ø¿¡°Ô ¿¬±¸ ¼¾ÅÍ¿¡ ÀÇÇÏ¿© ºñ¹Ð·Î ¾çµµµÉ °ÍÀÓÀ» ¼ö¶ôÇÕ´Ï´Ù. º»ÀÎÀº ¿µ»ó ¿¬±¸ ÀýÂ÷ ¹×/¶Ç´Â ÀÚ·áÀÇ °ËÁõÀ» À§ÇÏ¿©, ÀÇ·ÚÀÚ°¡ ºñ¹ÐÀ¯Áö¸¦ À§¹ÝÇÏÁö ¾ÊÀ¸¸é¼­, ¿øº» ÀÇ·á ±â·Ï¿¡ ´ëÇÏ¿© Á÷Á¢ÀûÀ¸·Î Á¢±ÙÇÏµµ·Ï Çã°¡µÉ °ÍÀÓÀ» µ¿ÀÇÇÕ´Ï´Ù. 
● º»ÀÎÀº ¾ðÁ¦¶óµµ ºÒÀÌÀÍ ¾øÀÌ ¿¬±¸¸¦ Áß´ÜÇÒ ¼ö ÀÖÀ½À» ¾Ë°í ÀÖ½À´Ï´Ù. ±×·¯³ª, ´ã´çÀÇ»ç°¡ ¿¬±¸ Á¾·á ½Ã¿¡ ±ÍÇÏÀÇ °Ç°­ »óÅÂ¿¡ ´ëÇØ º¸°íÇÒ ¼ö ÀÖ°í, ±ÍÇÏ, ±ÍÇÏÀÇ ÁÖÄ¡ÀÇ ¶Ç´Â Ä£Ã´¿¡°Ô ¿¬¶ôÇÏ´Â µî ¸ðµç ÀûÀýÇÑ ¼ö´ÜÀ¸·Î ÀÌ·¯ÇÑ Á¤º¸¸¦ ¼öÁýÇÏ´Â °ÍÀÌ ÀÌ ¿¬±¸ °á°úÀÇ ÇØ¼®¿¡ Áß¿äÇÏ´Ù´Â °ÍÀ» ¾Ë°í ÀÖ½À´Ï´Ù. 
● ÀÌ ¿¬±¸ÀÇ ¿ä±¸ »çÇ×¿¡¼­, º»ÀÎÀº ÀÎÁ¾Àû Ç÷Åë¿¡ °üÇÑ °ÍÀ» Æ÷ÇÔÇÏ¿© ÀÌ ¿¬±¸ Áß ¼öÁý µÈ ÀÚ·áµéÀÌ ÀÇ·ÚÀÚ ¹× ÀÇ·ÚÀÚ¸¦ ´ë½ÅÇÏ¿© Àü»ê ½Ã½ºÅÛÀ¸·Î °¡°ø Ã³¸®µÉ ¼ö ÀÖ´Ù´Â °Í¿¡ µ¿ÀÇÇÕ´Ï´Ù. º»ÀÎÀº ¹ý¿¡ µû¶ó¼­ ¿¬±¸ÀÚ¿Í ÇÔ²² ¾î´À ¶§µç ÀÌ ÀÚ·áµé¿¡ Á¢±ÙÇÏ¿© ¼öÁ¤ÇÒ ¼ö ÀÖ´Â ±Ç¸®¸¦ Çà»çÇÒ ¼ö ÀÖ½À´Ï´Ù. 
● º»ÀÎÀº ¼º¸í ¶Ç´Â ¿¬±¸ Âü°¡ÀÚ·Î½á º»ÀÎÀ» È®ÀÎÇÒ ¸¸ÇÑ ¾î¶² ÀÚ·áµµ, °ü°è ±ÔÁ¤¿¡¼­ ¿ä±¸µÇ´Â °æ¿ì¸¦ Á¦¿ÜÇÏ°í´Â, º»ÀÎÀÇ ¼­¸é Çã°¡ ¾øÀÌ´Â °ø°³µÇÁö ¾ÊÀ» °ÍÀÔ´Ï´Ù. 
● º»ÀÎÀº ÀÇ·á ±â¿Õ·Â¿¡ °üÇÑ ¸ðµç Áú¹®¿¡ ´ëÇÏ¿© Á¤Á÷ÇÏ°Ô ´äº¯ÇÏ¿´°í, ´ã´çÀÇ»ç°¡ º»ÀÎ¿¡°Ô ºÎ°úÇÏ°í ÇÇÇèÀÚ ¼³¸í¼­¿¡ ³ª¿­µÈ, ¸ðµç ±ÔÀ²°ú ±ÔÁ¤À» ÁØ¼öÇÒ °ÍÀÓÀ» ¼±¾ðÇÕ´Ï´Ù. 
● ¼­¸í ÈÄ, º»ÀÎÀº ¼­¸éÀ¸·Î µÈ ÇÇÇèÀÚ µ¿ÀÇ¼­ »çº»À» ¹ÞÀ» °ÍÀÔ´Ï´Ù. 
2012³â ¿ù ÀÏ

ÇÇÇèÀÚ(»ý³â¿ùÀÏ/¼º¸í/ÀÚÇÊ ¼­¸í/³¯ÀÎ):
Ä£Á· º¸È£ÀÚ(ÇÇÇèÀÚ¿ÍÀÇ °ü°è/¼º¸í/ÀÚÇÊ ¼­¸í/³¯ÀÎ):
¿¬±¸ Ã¥ÀÓÀÚ(¼º¸í/ÀÚÇÊ ¼­¸í/ÀüÈ­ ¹øÈ£):
µ¿ÀÇ ±¸µæÀÚ(¼º¸í/ÀÚÇÊ ¼­¸í/ÀüÈ­ ¹øÈ£):

¸¸ÀÏ ±ÍÇÏ²²¼­ ÀÌ ¿¬±¸³ª ÇÇÇèÀÚ·Î¼­ ±ÍÇÏÀÇ ±Ç¸®¿¡ °üÇÏ¿© ¹®ÀÇÇÏ½Ç °ÍÀÌ ÀÖ°Å³ª ¶Ç´Â ¿¬±¸¿Í °ü·ÃÇÏ¿© ¼Õ»óÀ» ÀÔÀ¸¼ÌÀ» °æ¿ì¿¡´Â ¿¬±¸ Ã¥ÀÓÀÚ ÀÌ´öÈñ(053)420-¿¡°Ô ¿¬¶ôÇÏ¿© ÁÖ½Ê½Ã¿À.

´ç´¢ È¯ÀÚ¿¡¼­ÀÇ ½ÄÀÌ¿ä¹ý È¿°ú¿¡ °üÇÑ ¿¬±¸ ¼³¹®Áö

1. ¼º¸í                        Â÷Æ®¹øÈ£               

2. ÁÖ¹Îµî·Ï »ý³â¿ùÀÏ
  ¡à¡à¡à¡à³â ¡à¡à¿ù ¡à¡àÀÏ  ¸¸ ³ªÀÌ (   ¼¼)

3. ¿¬¶ôÃ³ (¢Î      -      -      , ÈÞ´ëÀüÈ­      -      -      )

4. ÁÖ¼Ò                                                                

¿îµ¿ ¹× ½ÅÃ¼È°µ¿

1. ÃÖ±Ù 1ÁÖÀÏ µ¿¾È Æò¼Òº¸´Ù ¸öÀÌ ¸Å¿ì Èûµé°Å³ª ¼ûÀÌ ¸¹ÀÌ °¡»Û °Ý·ÄÇÑ ½ÅÃ¼È°µ¿À» 10ºÐÀÌ»ó Çß´ø ³¯Àº ¸çÄ¥ÀÔ´Ï±î?   ¡àÀÏ
¡Ø °Ý·ÄÇÑ(°í°­µµ) ½ÅÃ¼È°µ¿(¿¹½Ã)
 ´Þ¸®±â(Á¶±ë), µî»ê, ºü¸¥ ¼Óµµ·Î ÀÚÀü°Å Å¸±â, ºü¸¥ ¼ö¿µ,  ÁÙ³Ñ±â, ½ºÄõ½Ã, Å×´Ï½º µî	

     ¡æ 1-1 ÀÌ·¯ÇÑ °Ý·ÄÇÑ ½ÅÃ¼È°µ¿À» ÇÑ ³¯, º¸Åë ÇÏ·ç¿¡ ¸î ºÐ°£ Çß½À´Ï±î?
        ÇÏ·ç¿¡ ¡à¡à½Ã°£ ¡à¡àºÐ

2. ÃÖ±Ù 1ÁÖÀÏ µ¿¾È Æò¼Òº¸´Ù ¸öÀÌ Á¶±Ý Èûµé°Å³ª ¼ûÀÌ ¾à°£ °¡»Û Áßµîµµ ½ÅÃ¼È°µ¿À» 10ºÐ ÀÌ»ó Çß´ø ³¯Àº ¸çÄ¥ÀÔ´Ï±î?(´Ü, °È±â´Â Á¦¿ÜÇÕ´Ï´Ù.)    ¡àÀÏ
¡Ø Áßµîµµ(Áß°­µµ) ½ÅÃ¼È°µ¿(¿¹½Ã)
 ÃµÃµÈ÷ ÇÏ´Â ¼ö¿µ, º¹½ÄÅ×´Ï½º, ¹è±¸, º£µå¹ÎÅÏ, Å¹±¸, °¡º­¿î ¹°°Ç ³ª¸£±â µîÀÇ Á÷¾÷ È°µ¿ ¹× Ã¼À° È°µ¿, 	

     ¡æ 2-1 ÀÌ·¯ÇÑ Áßµîµµ ½ÅÃ¼È°µ¿À» ÇÑ ³¯, º¸Åë ÇÏ·ç¿¡ ¸îºÐ°£ Çß½À´Ï±î?
        ÇÏ·ç¿¡ ¡à¡à½Ã°£ ¡à¡àºÐ

3. ÃÖ±Ù 1ÁÖÀÏ µ¿¾È ÇÑ¹ø¿¡ Àû¾îµµ 10ºÐ ÀÌ»ó °É¾ú´ø ³¯Àº ¸çÄ¥ÀÔ´Ï±î? ¡àÀÏ
¡Ø ÃâÅð±Ù ¹× µîÇÏ±³, ÀÌµ¿ ¹× ¿îµ¿À» À§ÇØ °È´Â °ÍÀ» ¸ðµÎ Æ÷ÇÔÇÏ¿© ´ë´äÇØ ÁÖ½Ê½Ã¿À.	

¡Ø ÃâÅð±Ù ¶Ç´Â µîÇÏ±³, ÀÌµ¿ ¹× ¿îµ¿À» À§ÇØ °È´Â °ÍÀ» ¸ðµÎ Æ÷ÇÔÇÏ¿© ´ë´äÇØ ÁÖ½Ê½Ã¿À.
     ¡æ 3-1 ÀÌ·¯ÇÑ ³¯ Áß ÇÏ·ç µ¿¾È °È´Â ½Ã°£Àº º¸Åë ÇÏ·ç¿¡ ¸î ºÐ°£ Çß½À´Ï±î?
        ÇÏ·ç¿¡ ¡à¡à½Ã°£ ¡à¡àºÐ

4. ÃÖ±Ù 1ÁÖÀÏ µ¿¾È ³·¿¡ ´©¿ö°è½Ã°Å³ª ¾É¾Æ°è½Ã´Â ½Ã°£ÀÌ ÇÏ·ç Æò±Õ ¾ó¸¶³ª µË´Ï±î? 
¡Ø TV¸¦ º¸±âÀ§ÇØ¼­ ´©¿ö°è½Ã°Å³ª ³·Àá ½Ã°£±îÁö¸¦ ¸ðµÎ Æ÷ÇÔÇÏ¿© ´ë´äÇØ ÁÖ½Ê½Ã¿À.	

     ¡æ ÇÏ·ç¿¡ ¾à ¡à¡à½Ã°£ ¡à¡àºÐ   


Case record form(Áõ·Ê ±â·ÏÁö)


1. ±âº» Á¤º¸
ÀÓ»ó ½ÃÇè±º	¡à´ç´¢º´ ½ÄÀÌ±º     ¡àÇö¹Ì Ã¤½Ä±º	
È¯ÀÚ ¹øÈ£		
È¯ÀÚ ÀÌ¸§		
³ªÀÌ		
´ç´¢º´ Ã¹ Áø´Ü¿¬·É		
µ¿¹Ý»óº´	¡à°íÇ÷¾Ð   ¡à°íÁöÇ÷Áõ   ¡à±× ¿Ü ÁúÈ¯ :                            	


2. Medication (¡Ø±âÁØ ½ÃÁ¡: µ¿ÀÇ¼­ Ãëµæ ³¯Â¥)
(1) DM Medication 
¨ç ÀÎ½¶¸° Ä¡·á
  Ⓐ Glargine  Ⓑ Premixed insulin  Ⓒ NPH  Ⓓ Glargine + rapid-acting analog
¨è Metformin
¨é Sulfonylurea
¨ê Thiazooidinedione
¨ë º¹ÇÕ Ä¡·á                                                   
¨ì ±âÅ¸ ´ç´¢ medication                                                   

(2) DM ¿ÜÀÇ Medication 
¨ç Ç÷¾Ð Á¶Àý¾à                                                  
¨è ÁöÁú Á¶Àý¾à                                                  
¨é ±× ¿Ü º¹¿ëÁßÀÎ ¾à                                                  

(3) Postmenopausal hormone º¹¿ë ¿©ºÎ :  ¡àÇöÀç º¹¿ëÁß   ¡àÇöÀç º¹¿ëÇÏÁö ¾ÊÀ½

3. È¯ÀÚÀÇ ±âº» DM ÇÕº´Áõ
¨ç Eye involvement 
¨è Renal involvement 
¨é Neuropathy (¡Ø¾Æ·¡ Ç×¸ñ¿¡ Ã¼Å© ÈÄ ÃÑÁ¡ ±âÀç)

¡Ø ´ÙÀ½Àº ±ÍÇÏÀÇ ´Ù¸®¿Í ¹ß¿¡ ³ªÅ¸³ª´Â ´À³¦À» Á¶»çÇÏ´Â Ç¥ÀÔ´Ï´Ù. Àß »ý°¢ÇÏ¿©, Æò¼Ò ´À³¦ÀÌ ¾î¶²Áö ¡®¿¹¡¯ ¶Ç´Â ¡®¾Æ´Ï¿À¡¯·Î Ç¥½ÃÇØ ÁÖ½Ã±â ¹Ù¶ø´Ï´Ù.
Ã¼Å©¸®½ºÆ®	¿¹	¾Æ´Ï¿À	
(1) ¹ß ¶Ç´Â ´Ù¸®¿¡ °¨°¢ÀÌ ¾ø½À´Ï±î?			
(2) ¹ß ¶Ç´Â ´Ù¸®¿¡ È­²ö°Å¸®´Â ÅëÁõÀ» ´À³¤ ÀûÀÌ ÀÖ½À´Ï±î?       			
(3) ¹ß¿¡ ¹«¾ùÀÌ ´êÀ» ¶§ °ú¹ÎÇÏ°Ô ´À³§´Ï±î? 			
(4) ¹ß ¶Ç´Â ´Ù¸®¿¡ °©ÀÚ±â Áã°¡ ³³´Ï±î?			
(5) ¹ß ¶Ç´Â ´Ù¸®¿¡ Âî¸£´Â µíÇÑ ´À³¦À» ¹ÞÀº ÀûÀÌ ÀÖ½À´Ï±î?			
(6) ÀÌºÒÀÌ ÇÇºÎ¿¡ ´êÀ» ¶§ ¾ÆÇÄÀ» ´À³§´Ï±î?			
(7) ¸ñ¿åÇÒ ¶§, ¶ß°Å¿î ¹°°ú Â÷°¡¿î ¹°À» ±¸ºÐÇÒ ¼ö ¾ø½À´Ï±î?			
(8) ¹ß¿¡ ±îÁø »óÃ³°¡ »ý±ä ÀûÀÌ ÀÖ½À´Ï±î?			
(9) ÀÇ»ç·ÎºÎÅÍ ´ç´¢º´¼º ½Å°æº´ÁõÀÌ¶ó°í Áø´ÜµÈ ÀûÀÌ ÀÖ½À´Ï±î?			
(10) ´Ù¸®³ª ¹ß¿¡ ¸¶ºñ°¡ ÀÖ½À´Ï±î?			
(11) ´Ù¸®³ª ¹ßÀÇ Áõ»óÀÌ ¹ã¿¡ ´õ ½ÉÇØÁý´Ï±î?			
(12) °ÉÀ» ¶§ ´Ù¸®°¡ ¾ÆÇÅ´Ï±î?			
(13) °ÉÀ» ¶§ ¹ß¿¡ °¨°¢À» ´À³¥ ¼ö ¾ø½À´Ï±î?			
(14) ¹ßÀÇ ÇÇºÎ°¡ ³Ê¹« °ÇÁ¶ÇØ¼­ ÀÚÁÖ °¥¶óÁý´Ï±î?			
(15) ¹ßÀÌ³ª ¹ß°¡¶ôÀ» ÀÚ¸£´Â ¼ö¼úÀ» ¹ÞÀº ÀûÀÌ ÀÖ½À´Ï±î?			
ÇÕ°è 	(   /15Á¡)	


¨ê Other microvascular complication:                                
¨ë Macrovascular complication: ¡àÇãÇ÷¼º ½ÉÁúÈ¯    ¡à³úÁ¹Áß    ¡à ±× ¿Ü:       


4. ½ÅÃ¼°èÃø
	¿¬±¸ ½ÃÀÛ ½ÃÁ¡	4ÁÖ 	12ÁÖ 	
Å°(cm)				
¸ö¹«°Ô(Kg)				
Çã¸®µÑ·¹(cm)				
¾ûµ¢ÀÌµÑ·¹(cm)				
BMI(kg/m2)				
¼öÃà±âÇ÷¾Ð/ÀÌ¿Ï±âÇ÷¾Ð(mmHg)				


5. Lab °á°ú
	¿¬±¸ ½ÃÀÛ ½ÃÁ¡	4ÁÖ 	12ÁÖ 	
HbA1c(%)				
Fasting plasma glucose(mg/dL)				
Triglycerides(mg/dL)				
Total Cholesterol(mg/dL)				
HDL Cholesterol(mg/dL)				
GGT(U/L)				
Insulin				


µî·Ï½Ã ½ÃÇè¿¬±¸¿ø                   ¼­¸í                         ³â       ¿ù       ÀÏ

Ã¥ÀÓ ¿¬±¸ÀÚ                         ¼­¸í                         ³â       ¿ù       ÀÏ
